# Spinal Cord Injury Changes the Structure and Functional Potential of Gut Bacterial and Viral Communities

**DOI:** 10.1128/mSystems.01356-20

**Published:** 2021-05-11

**Authors:** Jingjie Du, Ahmed A. Zayed, Kristina A. Kigerl, Kylie Zane, Matthew B. Sullivan, Phillip G. Popovich

**Affiliations:** a Department of Microbiology, The Ohio State University, Columbus, Ohio, USA; b Department of Neuroscience, The Ohio State University College of Medicine, Columbus, Ohio, USA; c The Belford Center for Spinal Cord Injury, The Ohio State University College of Medicine, Columbus, Ohio, USA; d The Center for Brain and Spinal Cord Repair, The Ohio State University College of Medicine, Columbus, Ohio, USA; e Medical Scientist Training Program, The Ohio State University College of Medicine, Columbus, Ohio, USA; f Department of Civil, Environmental and Geodetic Engineering, The Ohio State University, Columbus, Ohio, USA; g Infectious Disease Institute, The Ohio State University, Columbus, Ohio, USA; h Center of Microbiome Science, The Ohio State University, Columbus, Ohio, USA; University of Copenhagen

**Keywords:** gut dysbiosis, metagenomics, microbiome, spinal cord injury, virome

## Abstract

Emerging data indicate that gut dysbiosis contributes to many human diseases, including several comorbidities that develop after traumatic spinal cord injury (SCI). To date, all analyses of SCI-induced gut dysbiosis have used 16S rRNA amplicon sequencing. This technique has several limitations, including being susceptible to taxonomic “blind spots,” primer bias, and an inability to profile microbiota functions or identify viruses. Here, SCI-induced gut dysbiosis was assessed by applying genome- and gene-resolved metagenomic analysis of murine stool samples collected 21 days after an experimental SCI at the 4th thoracic spine (T4) or 10th thoracic spine (T10) spinal level. These distinct injuries partially (T10) or completely (T4) abolish sympathetic tone in the gut. Among bacteria, 105 medium- to high-quality metagenome-assembled genomes (MAGs) were recovered, with most (*n* = 96) representing new bacterial species. Read mapping revealed that after SCI, the relative abundance of beneficial commensals (Lactobacillus johnsonii and *CAG-1031* spp.) decreased, while potentially pathogenic bacteria (Weissella cibaria, Lactococcus lactis*_A,* Bacteroides thetaiotaomicron) increased. Functionally, microbial genes encoding proteins for tryptophan, vitamin B_6_, and folate biosynthesis, essential pathways for central nervous system function, were reduced after SCI. Among viruses, 1,028 mostly novel viral populations were recovered, expanding known murine gut viral species sequence space ∼3-fold compared to that of public databases. Phages of beneficial commensal hosts (*CAG-1031, Lactobacillus*, and *Turicibacter*) decreased, while phages of pathogenic hosts (*Weissella, Lactococcus*, and class *Clostridia*) increased after SCI. Although the microbiomes and viromes were changed in all SCI mice, some of these changes varied as a function of spinal injury level, implicating loss of sympathetic tone as a mechanism underlying gut dysbiosis.

**IMPORTANCE** To our knowledge, this is the first article to apply metagenomics to characterize changes in gut microbial population dynamics caused by a clinically relevant model of central nervous system (CNS) trauma. It also utilizes the most current approaches in genome-resolved metagenomics and viromics to maximize the biological inferences that can be made from these data. Overall, this article highlights the importance of autonomic nervous system regulation of a distal organ (gut) and its microbiome inhabitants after traumatic spinal cord injury (SCI). By providing information on taxonomy, function, and viruses, metagenomic data may better predict how SCI-induced gut dysbiosis influences systemic and neurological outcomes after SCI.

## Abstract (precis)

To our knowledge, this is the first article to apply metagenomics to characterize changes in gut microbial population dynamics caused by a clinically relevant model of central nervous system (CNS) trauma. It also utilizes the most current approaches in genome-resolved metagenomics and viromics to maximize the biological inferences that can be made from these data.

## INTRODUCTION

Gut microbiota protect mammals from pathogen colonization (reviewed in reference 1), regulate gut permeability (2), stimulate the immune system (reviewed in reference 1), synthesize essential vitamins (reviewed in reference 3), produce secondary bile acids (4, 5), produce short-chain fatty acids (SCFAs), and provide metabolic fuel for colonocytes by breaking down indigestible food sources (6, 7). Gut microbes are also key components of the “brain-gut axis,” i.e., the bidirectional system of communication between the central nervous system (CNS) and the digestive system. Gut microbes have been shown to be essential for normal CNS development, functioning, and recovery after injury (8–11) and for regulating host neural activity and behavior in response to environmental cues (12). Indeed, gut-derived microbes produce various neuroactive metabolites or precursor molecules (e.g., tryptophan), which are needed to synthesize serotonin, dopamine, gamma-aminobutyric acid (GABA), acetylcholine, and melatonin (13). These neuroactive metabolites signal the CNS via vagal afferents, or they enter the circulation and pass directly into the neural parenchyma across the blood-brain barrier (14, 15). Gut microbes are also capable of indirectly signaling the CNS by influencing innate and adaptive immunity; and the immune system, like the gut, exerts bidirectional communication with the CNS (reviewed in reference 16).

Homeostasis of the gastrointestinal (GI) tract, including microbial homeostasis, is dependent on the enteric nervous system of the GI tract, which is innervated by the parasympathetic efferents from the vagus nerve and sacral spinal cord, presympathetic nerves originating in the brainstem, and sympathetic efferents originating exclusively from the spinal cord (17, 18). When the spinal cord is injured, axons that normally descend from the brain/brainstem to control spinal sympathetic neurons are lost or damaged (17). Consequently, after spinal cord injury (SCI), normal sympathetic control of the small bowel and colon is lost, leading to impaired gut motility, mucosal secretions, vascular tone, and immune function (19). Loss or disruption of one or more of these GI functions after SCI can disrupt the ecological balance of microorganisms in the gut, causing dysbiosis (20, 21). Indeed, lasting changes in gut bacterial composition have been documented in multiple clinical and preclinical studies of SCI (22–27). Disruption of this gut microbial ecosystem has been linked to various comorbidities that develop after SCI, such as metabolic disease, immune dysfunction, and mental and cognitive impairment (28–31). Unfortunately, because all published reports of SCI-induced gut dysbiosis have used 16S rRNA amplicon sequencing to characterize compositional changes in gut bacteria, reliable predictions about microbiota function remain elusive, as do data describing SCI-induced compositional changes in novel microbial species, including viruses (21).

Because viruses lack universal marker genes for taxonomic assignment, the genetic diversity of the gut virome remains largely unknown (32, 33). As the most abundant members of the enteric virome (34), bacteriophages may affect human health and disease by dramatically shaping gut bacterial communities and their functions. This can occur through predator-prey dynamics (reviewed in reference 35) and horizontal gene transfer (36) or by direct interactions between viruses and the immune system (37–40), even including a phage-mediated, non-host-derived immunity to protect against invading pathogens (41). There may or may not be a “healthy gut virome” (34, 42–44), but it is clear that individuals have unique, persistent viromes (42, 43). In the context of disease, disease-specific virome changes have been observed for inflammatory bowel disease (IBD) (45), ulcerative colitis (46), autism spectrum disorders (ASD) (47), colorectal cancer (48), type 1 diabetes (49, 50), and type 2 diabetes (51). However, it is notable that rather than considering all taxa as is done in environmental studies (52–54), most gut virome studies have limited their analyses to known taxonomy or missed the linkage of viruses to their hosts (55, 56). Nothing is yet known about how the gut virome is affected by SCI.

All forms of SCI examined to date cause gut dysbiosis (19, 22–27), but what remains unclear is whether SCI-induced changes in microbial population dynamics and the functional implications of those changes vary as a function of spinal injury level and/or injury severity. To expand upon current knowledge of SCI-induced gut dysbiosis, we use metagenomics to examine ecological and functional changes in gut bacterial and viral communities after SCI in a murine model, controlling for diet and antibiotic exposure (common confounders for microbiome studies) (22, 23, 57). Ecological and functional changes in the gut microbiota were compared in mice receiving SCI at the 4th thoracic spine (T4) or 10th thoracic spine (T10) spinal levels. We show that the severity of gut dysbiosis is affected by spinal injury level; more robust changes were noted when SCI occurred at high spinal levels (T4), which causes greater imbalance in autonomic tone in the gut. Our novel data also reveal SCI-dependent changes in the microbiome and virome, as well as related metabolic pathways, providing a myriad of new hypotheses to guide future SCI studies.

## RESULTS AND DISCUSSION

### The composition and magnitude of change in gut microbiota caused by SCI vary as a function of spinal injury level.

**FIG 1:**
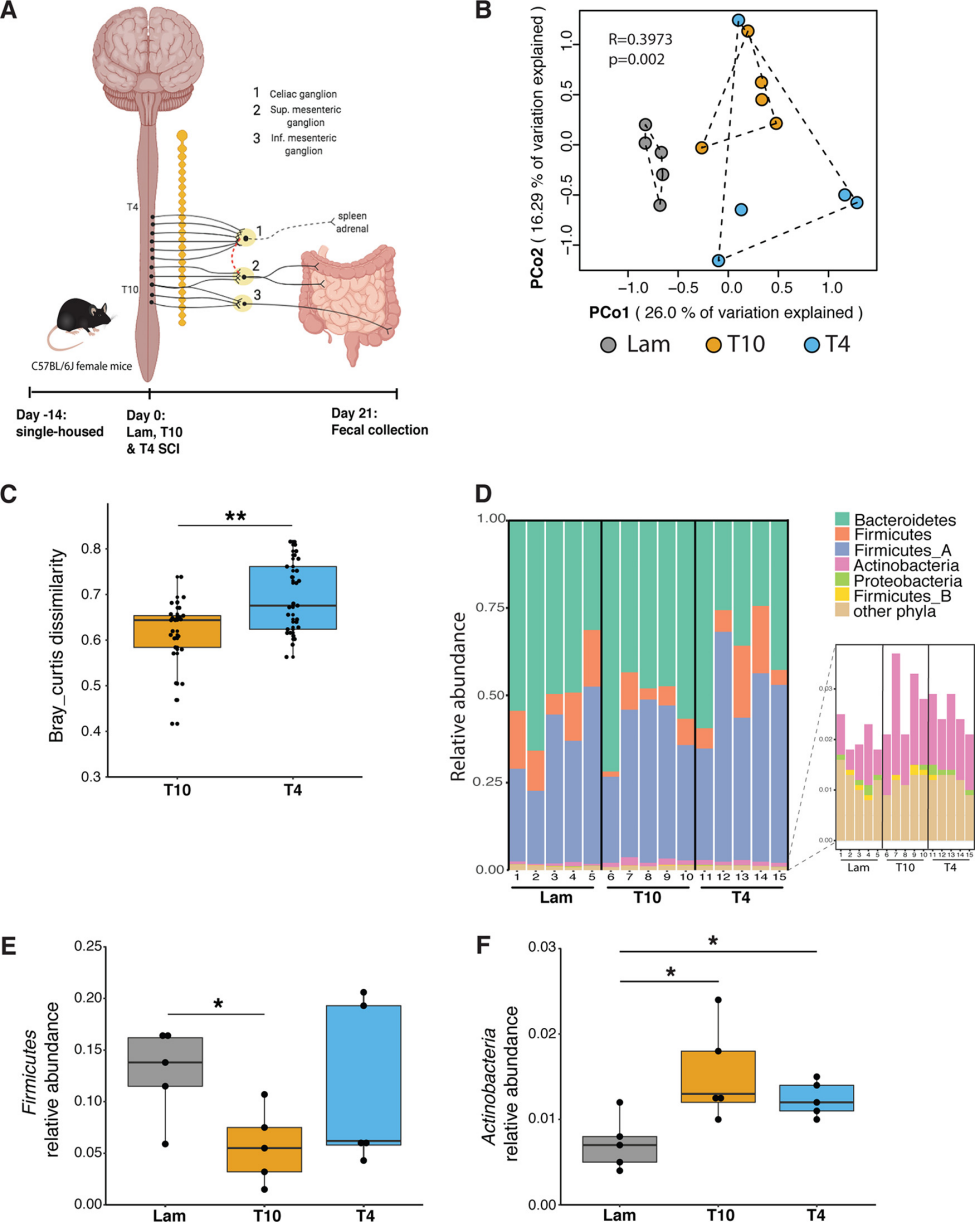
Intestinal microbial community composition was disturbed after spinal cord injury. (A) Fifteen mice were equally divided among three treatment groups: a sham surgery control group (Lam), an SCI group modeling injury at vertebral level T10, and an SCI group modeling injury at vertebral level T4. At 21 days postinjury, one fecal sample per mouse was collected for bulk microbiome isolation. (B) Principal-coordinate analysis (PCoA) of Bray-Curtis distances shows that microbial communities are different between the Lam, T4, and T10 groups (PERMANOVA, *R* = 0.3973, *P* = 0.002). Each data point indicates an individual mouse sample. (C) Box plot analysis showing the Bray-Curtis dissimilarities between the control group (Lam) and T4 or T10 SCI microbial communities. Each data point represents one Bray-Curtis dissimilarity comparison between individual samples in each of the other groups (Lam versus T4/T10; *n* = 5 of one group were individually compared to *n* = 5 from another group for a total of 25 comparisons between the Lam and T4 or T10 groups, respectively). A higher score suggests a higher dissimilarity of different individuals between the Lam and SCI groups (T4/T10). **, *P* < 0.01 by Wilcoxon rank sum test with a false discovery rate (FDR) of <0.05 calculated by “fdr” in R. (D) Intestinal microbial community composition at the phylum level. On the *x* axis, the numbers 1 to 15 represent individual mice within each group. (E and F) Box plots showing the relative abundance of the phyla *Firmicutes* (E) and *Actinobacteria* (F). All box plots shown display the median and quartiles, with each dot in the box plot representing an individual mouse sample, and each group (Lam, T4, T10) contains five samples. Read-based estimates of relative abundances of microbial taxa (see Materials and Methods) were used for all the analyses displayed. *, *P* < 0.05 (by Wilcoxon rank sum test; FDR < 0.05).

We hypothesized that sympathetic nervous system control over proximal and distal intestines will vary as a function of spinal injury level, causing differences in intestinal function that will directly affect the composition of the gut microbiome. The sympathetic preganglionic neurons (SPNs) controlling the small and large intestines are located primarily in the intermediolateral cell column in thoracic spinal segments T5 to T10 (58–60). Therefore, most brain and brainstem control over spinal autonomic networks that innervate the gut are lost when SCI occurs at or above the T5 spinal level. When SCI occurs at lower spinal levels, some reflex control over spinal autonomic neurons remains intact, with the magnitude of intact circuitry varying as a function of injury level and severity. To date, there have been no controlled studies to assess how the gut microbiota responds to SCI when there is some or no preservation of executive control (from brain/brainstem) over spinal autonomic reflexes, although data from human subject research predicts that spinal-level-dependent differences do exist. Indeed, the composition of gut microbiota in individuals with cervical SCI was found to be distinct from that in people with thoracic or lumbar SCI (23). Here, to directly test the hypothesis that the composition and magnitude of change in SCI-induced gut dysbiosis varies as a function of spinal injury level, three groups of mice were prepared (*n* = 15 mice in total; *n* = 5/group). Sham-injured control mice underwent laminectomy surgery at vertebral level T4 without spinal cord injury (Lam controls). Mice in the remaining two groups received a severe crush injury of the spinal cord at either the T10 or T4 spinal level. Based on published data, these two distinct spinal injury levels either partially preserve (T10) or abolish (T4) sympathetic innervation of the gut (58–61). Because gut microbes are transferable among species that share the same habitat, all mice were singly housed throughout the study, starting 2 weeks before SCI. At 21 days postinjury (dpi), fecal samples were collected and then individually prepared for microbiome analysis (Fig. 1A). The 21-dpi time period was chosen because published data indicate that gut dysbiosis fully develops at this time postinjury (19).

To characterize microbial community composition in each group, microbial taxa were identified using read-based and assembly-based approaches (see Materials and Methods and Fig. S1 in the supplemental material). A set of 14 single-copy marker genes were directly detected from the metagenomic reads using Hidden Markov Model (HMM) profiles, allowing for higher taxonomic (strain-level) resolution than is achievable by 16S rRNA gene sequencing and also avoiding the copy number variation problem that limits abundance estimation in 16S rRNA gene sequencing (62). Abundance-based comparisons (principal-coordinate analysis [PCoA] of Bray-Curtis dissimilarities using ribosomal protein L2 [rplB]; see Materials and Methods) of all microbial taxa present within the microbiome of each mouse revealed that the SCI groups cluster separately from those of Lam controls (permutational multivariate analysis of variance [PERMANOVA] *R* = 0.3973, *P = *0.002), indicating that SCI, regardless of injury level, disrupts microbial community structure (Fig. 1B). When we compared the between-group Bray-Curtis dissimilarity of each SCI group (T4 or T10) to healthy controls (Lam), we found that gut dysbiosis is exacerbated in mice with high-level T4 SCI (Fig. 1C). While 27.6% of the microbial operational taxonomic units (OTUs) (clustered at 97% nucleotide identity using the ribosomal protein L2) were shared between the different experimental groups (Fig. S2A), most (81.3%) of the unshared OTUs were rare species (<0.01% relative abundance), which limits how confidently we can ascribe their presence/absence (Data Set S1, tab 1). There was no difference in Shannon’s *H* between treatments (Fig. S2B).

10.1128/mSystems.01356-20.1 DATA SET S1 Data set containing summary of OTUs, MAGS, phages, and KEGG functions as well as statistical values (*P* values and FDR) used to identify significance. The data set also contains information about viral clusters, viral contigs, and virulence factors. Download Data Set S1, XLSX file, 3.8 MB. Copyright © 2021 Du et al. 2021 Du et al. https://creativecommons.org/licenses/by/4.0/ This content is distributed under the terms of the Creative Commons Attribution 4.0 International license.

10.1128/mSystems.01356-20.2 FIG S1 Flow diagrams showing the bioinformatics workflow. (A) Assembly of contigs; (B) analysis of microbial community and construction of MAGs; (C) identification of viral populations. Download FIG S1, PDF file, 0.8 MB. Copyright © 2021 Du et al. 2021 Du et al. https://creativecommons.org/licenses/by/4.0/ This content is distributed under the terms of the Creative Commons Attribution 4.0 International license.

10.1128/mSystems.01356-20.3 FIG S2 Differential abundance analysis of bacteria across healthy and SCI animals. (A) Venn diagram of the number of shared and unshared bacterial OTUs in different groups. (B) Box plot analysis showing Shannon’s *H* of the microbial communities between the Lam, T4, and T10 groups. Shannon’s *H* is an index of diversity, and a higher Shannon’s *H* suggests higher diversity of bacterial strains in the communities. All box plots shown display the median and quartiles, with each dot in the box plot representing an individual mouse sample, and each group (Lam, T4, T10) contains five samples. NS, not statistically significant by Wilcoxon rank sum test. (C) Heat map showing Z-score-normalized relative abundances of bacterial genera. Differentially abundant bacterial genera (*P* < 0.05 by Wilcoxon signed-rank test, FDR < 0.05 calculated by the Benjamini and Hochberg method) in either of two groups are indicated in red. Each row representing a unique bacterial genus was Z-score normalized. Bacterial genera on the *y* axis are clustered using Euclidean distances using pheatmap in R. (D) Box plot depicting eight rare (<0.5%) bacterial genera that are altered after SCI and differentially abundant between Lam controls and either the T4 or T10 group. **, *P* < 0.01; *, *P* < 0.05 (by Wilcoxon rank sum test). A false discovery rate (FDR) of <0.05 calculated by the Benjamini and Hochberg method was used here. Download FIG S2, PDF file, 0.9 MB. Copyright © 2021 Du et al. 2021 Du et al. https://creativecommons.org/licenses/by/4.0/ This content is distributed under the terms of the Creative Commons Attribution 4.0 International license.

Focusing in from the overall community comparisons, we next looked at changes that occurred at the phylum level. Among comparisons of 6 phyla (>0.1% relative abundance), the relative abundances of only two phyla changed significantly. By following the most recent Genome Taxonomy Database (GTDB) classification (63) (see Materials and Methods), these phyla were the *Firmicutes*, which was less abundant in the T10 SCI group (Fig. 1D and E), and the *Actinobacteria*, which was more abundant in both T10 and T4 SCI groups (Fig. 1D and F) than in our control group. We note that GTDB taxonomy separates the *Firmicutes*, *Firmicutes_A*, and *Firmicutes_B* into three separate phyla, which may be confusing but is phylogenomically well supported. Increased *Actinobacteria* may be associated with intestinal inflammation after SCI, as it has been in other inflammatory conditions like IBD (64), obesity (65), and rheumatoid arthritis (RA) (66).

**FIG 2:**
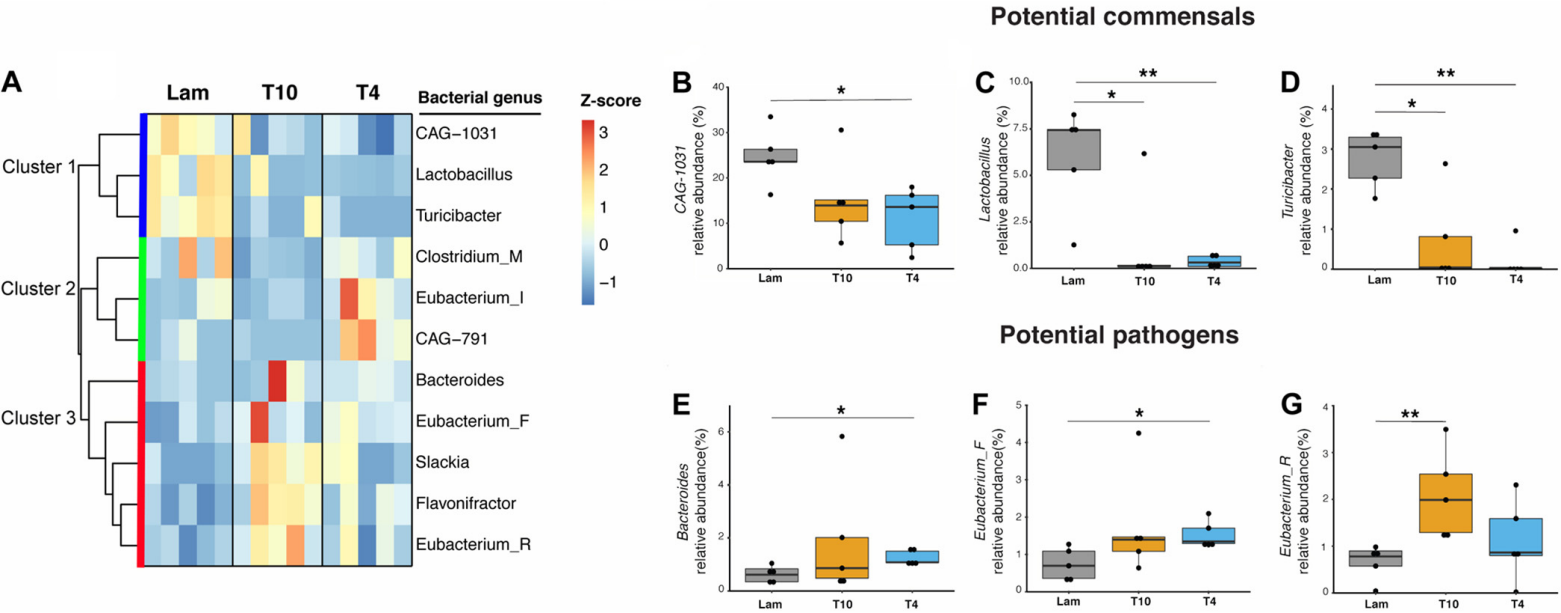
Genus-level bacterial abundances are altered after SCI. (A) Hierarchical clustering of differentially abundant bacterial genera (*P* < 0.05 by Wilcoxon rank sum test with a false discovery rate [FDR] of <0.05) shows three distinct clusters. Box plot analysis of select bacterial genera (with at least 0.5% relative abundance in any experimental group) indicates that *Lactobacillus*, *CAG-1031*, and *Turicibacter* (B to D) decreased after SCI, while *Bacteroides*, *Eubacterium_F*, and *Eubacterium_R* (E to G) increased after SCI in one injury level, compared to Lam controls. All box plots shown display the median and quartiles, with each dot in the box plot representing an individual mouse sample. Five individual mouse samples were used in each group. Read-based estimates of relative abundances of bacterial taxa (see Materials and Methods) were used for all the analyses displayed. **, *P* < 0.01; *, *P* < 0.05 (by Wilcoxon rank sum test). Analyses were done with an FDR of <0.05.

While relatively few phyla changed significantly between groups, there were many more changes at the genus level: 11 bacterial genera were differentially abundant (Wilcoxon rank sum test, *P < *0.05, false discovery rate [FDR] < 0.05) (Data Set S1, tab 2) between control and SCI groups (Fig. S2C, red text). Hierarchical clustering of these 11 bacterial genera identified three distinct clusters (Fig. 2A). Cluster 1 comprised genera that were less abundant in both SCI groups than in the Lam controls. Clusters 2 and 3 were composed of genera that had greater abundance in the T4 and T10 SCI groups, respectively, highlighting injury level-dependent effects on a subset of genera. Of these, one highly abundant taxon (>20% relative abundances in Lam), *CAG*-*1031*, was consistently less abundant in the T4 SCI group (Wilcoxon rank sum test, *P < *0.05, FDR < 0.05) (Fig. 2B). Two other abundant taxa (>1% relative abundance in Lam), *Lactobacillus* and *Turicibacter*, were less abundant in the T4 or T10 SCI groups (Wilcoxon rank sum test, *P < *0.05, FDR < 0.05) (Fig. 2C and D). *Turicibacter* spp. have been shown to promote host serotonin biosynthesis (67), while members of the *Lactobacillus* and *CAG*-*1031* genera are involved in key metabolic transformations in the gut (discussed below). A decrease in abundant commensals and probionts also could open niches for antagonistic commensals and pathobionts. Notably, seven taxa were more abundant in the T10 (*Eubacterium_R* and *Lachnospira*) or T4 (*Bacteroides*, *Weissella*, *Eubacterium_F*, *UBA9475*, and *Neglecta*) SCI groups than in controls (Fig. 2E to G; Fig. S2D). Most of these taxa, *Eubacterium_R*, *Lachnospira*, *Eubacterium_F*, *UBA9475*, and *Neglecta*, fall within the class *Clostridia* (Fig. 2F and G). Previously, using 16S RNA gene sequencing, we found that the *Clostridiales*, members of the class *Clostridia*, increase after SCI and that their relative abundance inversely correlated with recovery of motor function, suggesting that these microbes may adversely affect neurological function (19, 68).

Collectively, the above data suggest that gut dysbiosis is a consistent phenomenon after SCI but that greater disruption of sympathetic control over the colon, such as after high-level (T4) SCI, may drive distinct changes in the gut microbiota.

### Genome-centric view of SCI-induced changes of gut microbiota.

**FIG 3:**
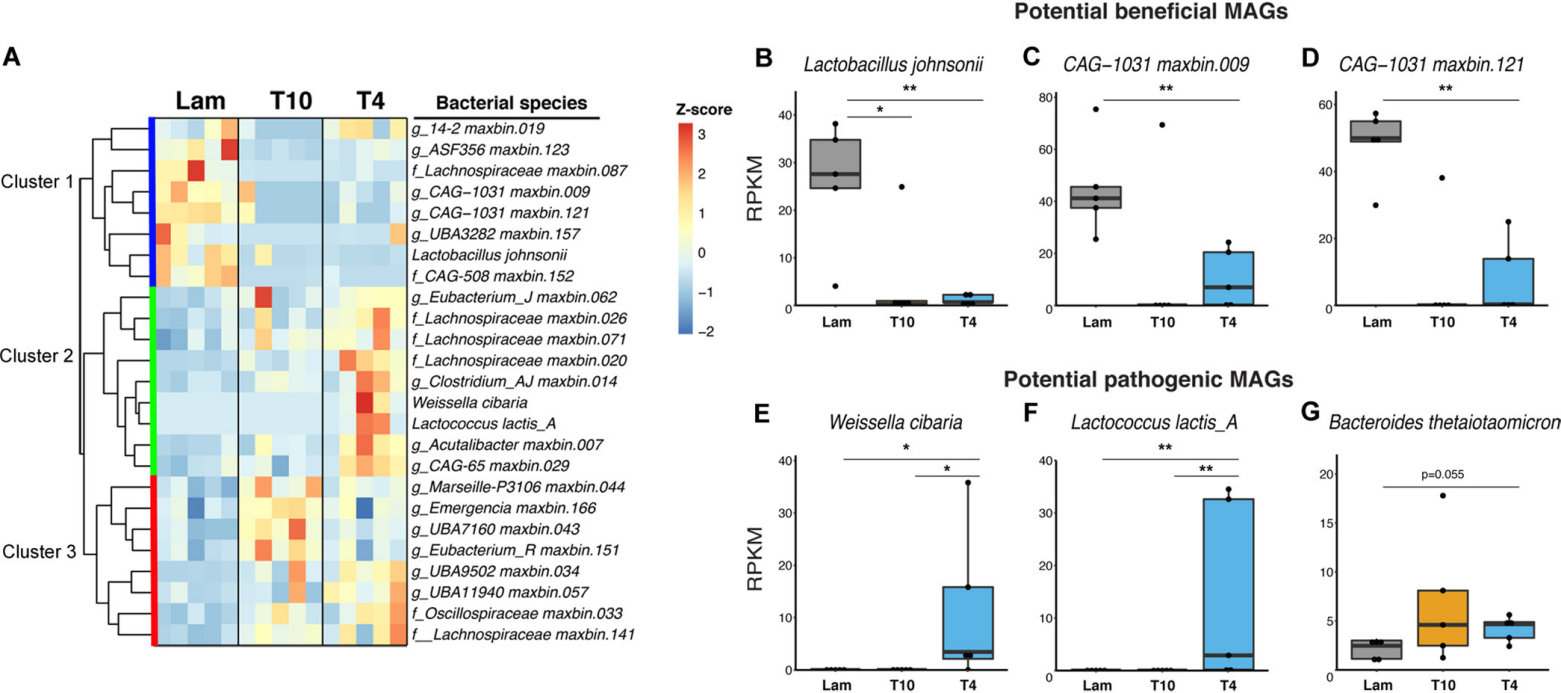
Species-level bacterial abundances are altered after SCI. (A) Hierarchical clustering of differentially abundant bacterial species (*P* < 0.05 by Wilcoxon rank sum test with a false discovery rate [FDR] of <0.05) shows three distinct clusters. Box plot analysis of select bacterial species shows that Lactobacillus johnsonii and two *CAG-1031* MAGs decreased after SCI (B to D), while Weissella cibaria, Lactococcus lactis*_A*, and Bacteroides thetaiotaomicron MAGs increased after SCI (E to G). All box plots shown display the median and quartiles, with each dot in the box plot representing an individual mouse sample. Five individual mouse samples are used in each group. All relative abundances shown here are represented as reads per kilobase per million mapped reads (RPKM; see Materials and Methods) of differentially abundant species-level MAGs. **, *P* < 0.01; *, *P* < 0.05 (by Wilcoxon rank sum test with an FDR of <0.05).

The above read-based analyses rely upon mapping to single-copy marker genes. At the community level, read-based analysis provides information for any microbes that have reference genomes available, and so read-based analyses are best used for assessing diversity patterns. However, metagenome-assembled genomes (MAGs) provide population genomes for the specific variants occurring in these samples as well as lineage-specific pathway- and gene-level information that can then be used to develop novel hypotheses regarding the functional capabilities and ecological niches for these microbes. Therefore, to further assess changing taxonomic patterns and infer microbial functions, we assembled the shotgun sequence data from each sample and then binned contigs to create *de novo* draft microbial genomes, i.e., MAGs. MAGs enable robust, genome-informed taxonomic classification within a sample and rather than predicting function from slow-evolving taxonomic marker genes, MAGs provide maps of ecologically relevant functional potential for known taxa (69). Using this approach, we recovered 112 MAGs (>60% complete, <10% contamination), including 105 MAGs of medium (*n* = 35; >70% complete, <10% contamination) to high (*n* = 70; >90% complete, <5% contamination) quality (see Materials and Methods) that recruited, on average, 54.7% of the quality-controlled sequencing reads (Data Set S1, tab 3). Most (*n* = 96) of these 105 MAGs represent previously unknown bacterial species as assessed against the GTDB-Tk v0.1.3 (63), the largest curated genomic taxonomy database available (69) (see Materials and Methods; Data Set S1, tab 4). Across the data set, 25 MAGs were differentially abundant between the Lam and SCI groups (Wilcoxon rank sum test, *P < *0.05, FDR < 0.05) (Fig. 3A; Fig. S3 and Data Set S1, tab 5).

10.1128/mSystems.01356-20.4 FIG S3 Species-level differential abundance analysis of bacteria across healthy and SCI animals. Heat map showing Z-score-normalized relative abundances of bacterial MAGs. Differentially abundant bacterial MAGs (*P* < 0.05 by Wilcoxon signed-rank test, FDR < 0.05 calculated by the Benjamini and Hochberg method) in either of two groups are indicated in red. Each row representing a unique bacterial MAG was Z-score normalized. Download FIG S3, PDF file, 0.5 MB. Copyright © 2021 Du et al. 2021 Du et al. https://creativecommons.org/licenses/by/4.0/ This content is distributed under the terms of the Creative Commons Attribution 4.0 International license.

**FIG 4:**
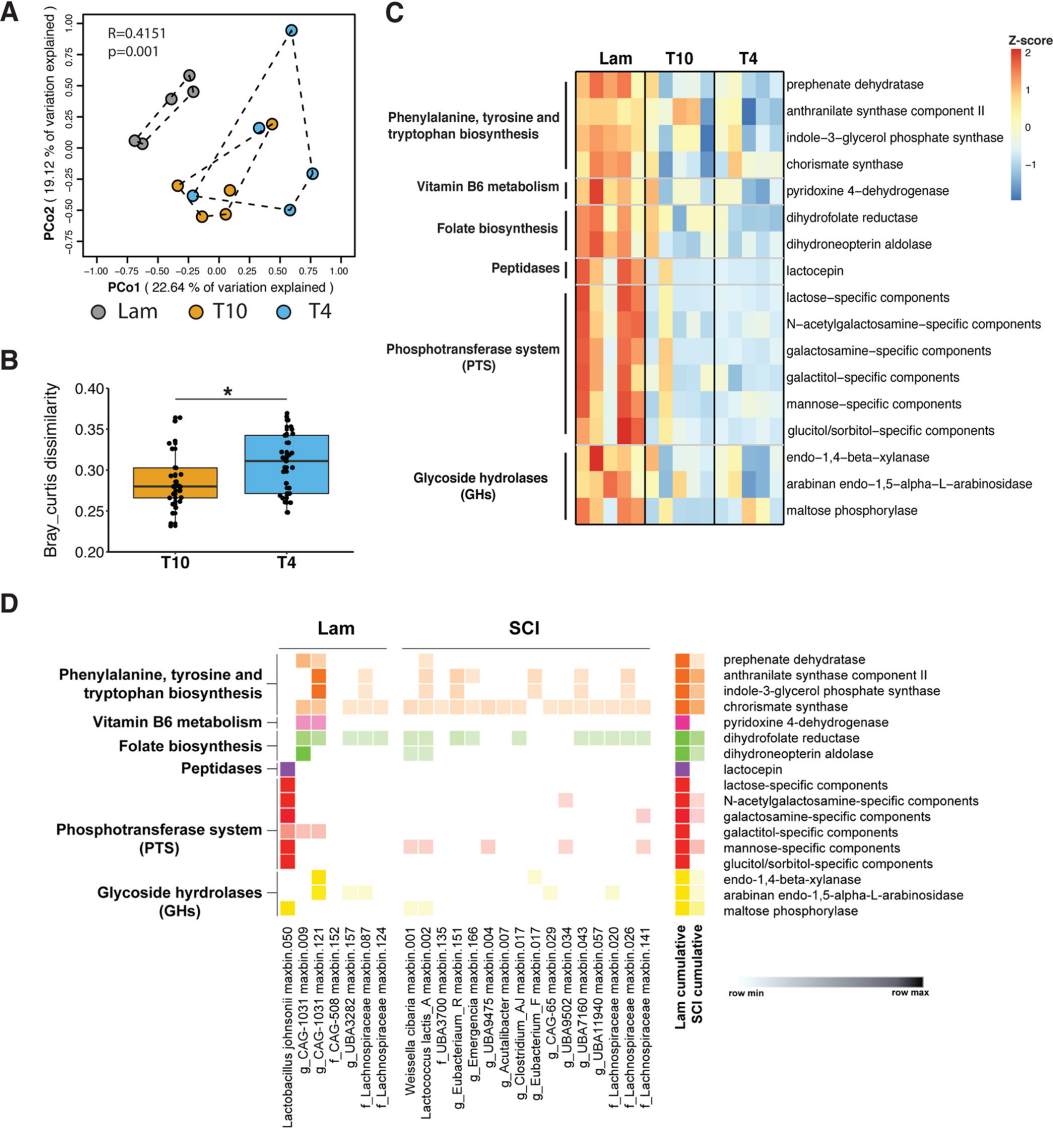
Predicted metabolic pathways are different between healthy and spinal cord injury animals. (A) Principal-coordinate analysis (PCoA) using Bray-Curtis distances shows that predicted protein clusters are different between the control group Lam and disease groups T4 and T10 (PERMANOVA, *R* = 0.4151, *P ≤ *0.001). Each data point indicates an individual mouse sample. (B) Box plot analysis showing the Bray-Curtis dissimilarities of predicted protein clusters between the control groups (Lam) and T4 or T10 SCI groups. Each data point represents one Bray-Curtis dissimilarity comparison between individual samples in each of the other groups (Lam versus T4/T10; *n* = 5 of one group were individually compared to *n* = 5 from another group for a total of 25 comparisons between the Lam and T4 or T10 groups, respectively). (C) Selected functions that are differentially abundant between groups (*P* < 0.05, by Wilcoxon rank sum test with a false discovery rate [FDR] of <0.05 calculated by “fdr” in R used for analysis). (D) MAG-resolved functional analysis for differentially abundant MAGs (*P* < 0.05 by Wilcoxon rank sum test, FDR < 0.05). The *x* axis shows the names of MAGs which are enriched in the Lam or T10/T4 groups, and the *y* axis shows the microbial functions from panel C and their predicted pathways. Only the MAGs with at least 80% completeness and less than 10% contamination are displayed here. The mean relative abundances (represented by reads per kilobase per million mapped reads [RPKM]) of KEGG functions of the differentially abundant MAGs were plotted (Morpheus; https://software.broadinstitute.org/morpheus/). A relative color scheme was used based on the minimum and maximum values in each row to convert values to colors.

Similar to what our analysis with single-copy marker genes showed at the genus level, hierarchical clustering of the 25 differentially abundant MAGS revealed 3 distinct clusters (Fig. 3A). Cluster 1 identifies bacterial species that were less abundant in SCI groups than in Lam controls, regardless of injury level. Cluster 2 species were of greater abundance only in the T4 high-level SCI group, and cluster 3 species were of greater abundance in both T4 and T10 SCI groups (Fig. 3A). These data again show that there are both injury-level-dependent and -independent changes in gut microbiota after SCI. Only one previously known species, Lactobacillus johnsonii, was significantly less abundant after both T4 and T10 SCI (Wilcoxon rank sum test, *P < *0.05, FDR < 0.05) (Fig. 3B). This species has widely been considered beneficial due to its anti-inflammatory effects (70–73) and its ability to metabolize inulin into a prebiotic that is further metabolized to short-chain fatty acids (SCFAs) like butyrate and propionate (74–77). Analysis of our L. johnsonii MAG revealed that it harbors the gene encoding the anti-inflammatory enzyme lactocepin (Fig. 4D; Data Set S1, tab 6) but not inulin metabolism genes, such as those coding for fructosyltransferases and fructansucrase (74–77). Since this MAG is only ∼85% complete, we cannot rule out that inulin metabolism genes are present in the native population, as others have reported (74–77), even though we did not detect it. Assuming that this MAG is functionally analogous, we hypothesize that after SCI, the dramatic difference in abundance (>8-fold) (Fig. 3B) of gut L. johnsonii markedly reduces beneficial SCFAs and impairs the immune regulatory properties of the gut microbiome. In support of this hypothesis, we previously reported that postinjury oral supplementation with a probiotic mixture of *Lactobacillus* and *Bifidobacterium* boosted T regulatory cells in the gut-associated lymphoid tissue (GALT) of SCI mice and that these changes were associated with improved locomotor recovery (19). In addition, fecal microbiota transplants restore SCFA levels after SCI and improve recovery (78).

Additionally, of the novel species that decreased, two belonged to the genus *CAG-1031* (family *Muribaculaceae*) and were represented by two high-quality (>90% completeness, <5% contamination) MAGs (Wilcoxon rank sum test, *P < *0.05, FDR < 0.05) (Fig. 3C and D). *CAG-1031* spp. decreased significantly with great magnitude (>5-fold) in T4 SCI mice, and though they decreased (>4-fold) in T10 mice, an outlier caused these changes to be not significant (Fig. 3C and D). Though there are no published *CAG-1031* functions, as a newly classified taxon under the latest GTDB update (69), members of the *Muribaculaceae* are known to synthesize folate and use diverse glycoside hydrolases to degrade complex polysaccharides (79). Indeed, the *CAG-1031* MAGs detected in our samples have the functional capacity to synthesize folate, break down complex polysaccharides, and synthesize vitamin B_6_ (Fig. 4D). The significant reduction in such abundant species after SCI suggests marked disruption in the gut microbiome’s capacity to metabolize complex carbohydrates and synthesize essential vitamins that cannot be made by mammalian host cells. Given their abundance, strong response, and plausible beneficial roles, we hypothesize that *CAG-1031* spp. are valuable probionts that, provided they can be grown in culture, might represent a novel probiotic therapy after SCI.

On the other hand, the relative abundances of three previously known bacterial species increased after SCI. Weissella cibaria and Lactococcus lactis*_A* MAGs were significantly enriched only in samples from T4 SCI mice (Wilcoxon rank sum test, *P < *0.05, FDR < 0.05) (Fig. 3E and F). Like L. johnsonii (above), *W. cibaria* and Lactococcus lactis*_A* are lactic acid producers (80, 81), and their expansion in the gut of T4 SCI mice may indicate the opportunistic growth of a species that was previously outcompeted by L. johnsonii and/or *CAG-1031* spp. In fact, both *W. cibaria* and Lactococcus lactis*_A* can act as opportunistic pathogens (80, 82), due to virulence factors such as hemolysins (80), which were found in our *W. cibaria* and Lactococcus lactis*_A* MAGs (Data Set S1, tab 7). B. thetaiotaomicron MAG also increased after SCI (Fig. 3G). B. thetaiotaomicron is the second most common agent in anaerobic Gram-negative infections in humans (83, 84) and is known to infect immune cells in a sulfatase-dependent manner and express proinflammatory lipooligosaccharides (LOS), which are analogous to the lipopolysaccharides (LPS) found in other Gram-negative bacterial families (85, 86). An analysis of our B. thetaiotaomicron MAG predicts that these bacteria carry lipopolysaccharide (LPS), polysaccharide, O-antigen biosynthesis genes, and multiple antibiotic resistance and sulfatase genes (Data Set S1, tab 7). Thus, an increase in B. thetaiotaomicron after SCI is expected to increase inflammation locally in the gut and also systemically via translocation.

Collectively, these data suggest that SCI decreases commensal abundances, which may open niches for antagonistic commensals and opportunistic pathogens that can increase inflammation and impair recovery after SCI. However, since most (>90%) of these MAGs represent novel species, the metagenomic approach provides an opportunity to establish baseline hypotheses beyond taxonomy about metabolic versatility—such as the vitamin B_6_, virulence factors, and LPS genes identified and described here—that could contribute to pathological comorbidities common in SCI individuals.

### SCI-induced gut dysbiosis is associated with loss of beneficial microbial functions.

Beyond the MAG-constrained analyses, we next sought to more broadly evaluate functional changes in the microbiome, since functions often vary more than taxonomy, both in human (87) and environmental systems (88). Previous work using 16S sequencing to characterize gut dysbiosis in a rat model of cervical SCI predicted differences in microbial functions between healthy and SCI rats using the PICRUSt algorithm (27). Although tools (i.e., PICRUSt) to infer microbial function from taxonomic profiling can be useful, they do not always correlate with results obtained using metagenomic sequencing (89), and critically, where correlations fail, these are likely to be niche-defining, ecologically relevant genes whose evolutionary histories (i.e., gene flow) are out of sync with slowly evolving ribosomal genes (89). Here, we translated predicted genes from assembled contigs (>500 bp in size) into amino acid sequences and then “organized” sequences into protein clusters. This eliminates bias against analyzing only proteins of known function (see Materials and Methods). As observed before at the taxon level (Fig. 1B), principal coordinate analysis (PCoA) revealed significant clustering of samples from the same group (PERMANOVA, *R* = 0.4151, *P ≤ *0.001) (Fig. 4A), with the largest separation occurring between the Lam and SCI groups. When the between-group Bray-Curtis dissimilarity of each SCI group (T4/T10) was compared with that of healthy controls (Lam), protein cluster changes were exacerbated in mice with high-level T4 SCI (Fig. 4B). Although several metabolic genes were differentially abundant between the T4 and T10 SCI groups (Fig. S4B and Data set S1, tabs 8 and 9), Shannon’s diversity index was not different between the SCI groups (Fig. S4A). These data indicate that SCI-induced disruption of gut microbial composition is accompanied by a corresponding change in microbial function and that these changes are exacerbated in mice with high-level T4 SCI.

10.1128/mSystems.01356-20.5 FIG S4 Predicted metabolic pathways that were differentially abundant between Lam controls and SCI groups. (A) Box plot analysis showing Shannon’s *H* of the protein clusters between the Lam, T4, and T10 groups. Shannon’s *H* is an index of diversity, and a higher Shannon’s *H* suggests a higher diversity of protein clusters in the communities. All box plots shown display the median and quartiles, with each dot in the box plot representing an individual mouse sample, and each group (Lam, T4, T10) contains five samples. NS, not statistically significant by Wilcoxon rank sum test. (B) Heat maps showing Z-score-normalized abundances of predicted KEGG functions, grouped by their different pathways. Only differentially abundant functions (*P* < 0.05 by Wilcoxon signed-rank test, FDR < 0.05) in either of the two groups are presented in this figure (Lam versus T4/T10). Red text indicates those KEGG functions that are significantly different between SCI injury level (T4 versus T10). Download FIG S4, PDF file, 0.4 MB. Copyright © 2021 Du et al. 2021 Du et al. https://creativecommons.org/licenses/by/4.0/ This content is distributed under the terms of the Creative Commons Attribution 4.0 International license.

To test the hypothesis that SCI impairs beneficial, microbially encoded metabolic functions in the gut, we performed both gene-based abundance comparisons on the whole microbial community (from the assembled contigs) and genome-based metabolic reconstructions (from the recovered MAGs). For gene-based analyses, all predicted functions in Lam controls were compared to those in SCI groups using the KEGG hierarchy to organize these functions into metabolic modules. Genes that were differentially abundant between the Lam and SCI groups (see Materials and Methods) fell into eight major metabolic pathways—carbohydrate, amino acid, lipid, vitamin, energy, glycan biosynthesis, enzymes involved in metabolism and other metabolisms, including biosynthesis of secondary metabolites, and xenobiotics biodegradation and metabolism (Wilcoxon rank sum test, *P < *0.05, FDR < 0.05) (Fig. S4 and Data Set S1, tab 8 and 9). After SCI, especially T4 SCI, we found significant derangement of gut microbial function, including lower abundance of many genes encoding components of the phosphotransferase system (PTS) and glycosyl hydrolases (GHs) (Fig. 4C; Fig. S4B). The PTS system controls sugar uptake by microbes; a reduction in PTS genes can impair microbial sugar utilization and their physiological functions. Many PTS genes, including those that encode the transport proteins specific for maltose, galactose, mannose, and lactose, were significantly reduced after SCI (compared to Lam). Conversely, mannitol PTS genes were significantly increased after T4 SCI (Fig. 4C; Fig. S4B). Utilization of fructose and glucose can decrease the abundance of beneficial commensals (90) and promote inflammation throughout the body (91). Conversely, utilization of lactose and mannose can decrease inflammation (92). Sugar alcohols like mannitol and sorbitol are more abundant in the mouse gut after antibiotic treatment, and enrichment of these sugar alcohols can promote the growth of the pathogen Clostridium difficile (93). Microbial GHs can break down indigestible food sources (e.g., fibers), producing neuroactive metabolites like short-chain fatty acids (94). In our study, genes encoding GHs that degrade xylan, arabinan, and maltose were significantly reduced after SCI compared to Lam (Fig. 4C; Fig. S4B).

Other microbial genes involved in regulating the biosynthesis of folate, vitamin B_6_, and amino acids (e.g., tryptophan, phenylalanine, tyrosine) also were reduced by SCI (Fig. 4C; Fig. S4B). In mammals, folate is essential for *de novo* pyrimidine synthesis, a prerequisite for making DNA. Folate also is critical for the maintenance of gastrointestinal health and neurological function (95, 96). In the context of SCI, recent data indicate that folate augments CNS repair and regeneration (97, 98). Vitamin B_6_ also plays a significant role in CNS, as it is required for the synthesis of key neurotransmitters, including epinephrine, dopamine, and serotonin (99, 100). Microbial synthesis of amino acids, notably tryptophan, supports gut barrier integrity and stimulates epithelial renewal. Tryptophan synthesized by microbes is converted to 5-hydroxytryptophan (5-HTP) by enterochromaffin cells in the gut and, when further metabolized to serotonin, influences gut motility (101, 102). 5-HTP also enters the circulation, where it can cross the blood-brain barrier and fuel serotonin synthesis in the brain and spinal cord (101–103). Decreased serotonin production has been linked to many disorders, including depression, anxiety, and irritable bowel syndrome (IBS), indicating that loss or a reduction in microbe-dependent tryptophan metabolism after SCI could be an undiagnosed cause or contributor to the higher-than-normal incidence of depression, fatigue, and anxiety in SCI individuals (104–107).

Finally, the microbial gene encoding lactocepin, an anti-inflammatory bacterial protease, was reduced after SCI. Lactocepin selectively degrades inflammatory chemokines, thereby reducing inflammation. Thus, a reduction in lactocepin would favor gut inflammation. In fact, in a murine colitis model, replenishing gut microbes that produce lactocepin effectively reduces gut pathology (108). After SCI, inflammatory cascades are initiated in the gut, and these can impair function of the enteric nervous system, which when combined with loss of normal autonomic tone to the gastrointestinal tract might exacerbate the consequences of a neurogenic bowel (19, 26, 109).

Next, using our *de novo* synthesized microbial reference genomes (MAGs), we mapped the functional changes shown in Fig. 4C (Data Set S1, tabs 9 and 10) to specific microbial species that were enriched significantly in Lam or SCI groups (Fig. 4D; Fig. S3). After these functional changes were mapped to specific MAGs, the mean relative abundance was plotted using a relative color scheme, showing that the cumulative effect of SCI is a reduction in many of these genes in comparison to Lam controls (Fig. 4D). Many MAGs, in Lam and SCI groups, contain genes controlling functions related to amino acids and secondary metabolite biosynthesis (chorismate synthase, indole-3-glycerol phosphate synthase, and anthranilate synthase component II). The consistency with which these genes were detected in most MAGs is not surprising, given that these genes are essential for building bacterial biomass, antibiotic production, and microbial community communication through quorum sensing (110–112). However, after SCI, some microbial functions were lost, mainly due to a loss or decrease in specific bacterial lineages (Fig. 4D; Data Set S1, tab 10). For example, the reduction in L. johnsonii after SCI leads to a decrease in lactocepin, PTS glucitol/sorbitol-specific components, galactitol-specific components, and lactose-specific components. Notably, the enzyme lactocepin is considered to be specific to *Lactobacillus* (108), and the abundance of lactocepin in our L. johnsonii MAG constitutes about 61.1% of the total lactocepin in the whole microbial community. In our analysis, lactocepin was also present in the less abundant *ASF356* MAG (Data Set S1, tab 11), revealing another candidate probiotic that could be mixed with L. johnsonii and our two novel *CAG-1031* spp. to collectively cover a large spectrum of microbial functions that are markedly reduced in the gut after SCI. Indeed, the consistent reduction in each of these bacterial species in the gut of SCI mice might enhance gut inflammation and reduce the ability of the microbiota to contribute to the metabolism and biosynthesis of key molecules required throughout the body. These results expand upon published data showing predicted functional changes caused by gut dysbiosis in SCI rats (27). Although SCI-induced changes were predicted in carbohydrate metabolism, bile acid biosynthesis, metabolism of cofactors and vitamins, and lipid biosynthesis in both mouse and rat SCI models, the metagenomic sequencing data in the current report provided additional insight. Specifically, we could map specific gene changes to MAGs that were differentially abundant between Lam and SCI groups (Fig. 4D). As a result, it is now possible to develop novel hypotheses about mechanisms ascribed to particular taxa that may be responsible for any observed functional changes. Such MAG-enabled hypotheses are ideal fodder for designing future interventional studies.

Taken together, these functional data support the hypothesis that SCI-induced gut dysbiosis promotes an inflammatory environment in the gut which could adversely affect gut motility and epithelial barrier integrity. This, in turn, has the potential to enhance bacterial translocation and systemic inflammation, exacerbating neuroinflammation and impairing neurological recovery after SCI. Recovery from SCI may be impaired further due to a reduction in the production and release of precursors needed for neurotransmitter synthesis and also vitamins and cofactors needed for optimal neurological function and CNS repair.

### SCI alters the gut virome.

Given the lack of marker genes for viruses (55), we used *de novo* assembly and population-based approaches to characterize the gut virome. These techniques are well established for characterizing viruses in the oceans and soils (53, 54, 113). In total, we recovered 2,675 viruses above 5 kb and 1,028 viral viruses above 10 kb from the bulk metagenomes. Specifically, population- or species-level taxa above 10 kb were discerned by clustering genomes at >95% average nucleotide identity genome-wide, and genus-level taxa were assigned using gene sharing networks. In total, this revealed 1,028 viral populations (approximately species-level taxonomy [54, 114, 115]; ≥10 kb) (see Materials and Methods), of which virtually all (*n* = 1,016; 98.8% of the 1,028) are novel species (Data Set S1, tab 12; see Materials and Methods). Indeed, exhaustive comparisons were made to public databases, including NCBI RefSeq v88 (4,061 complete genomes; >10 kb) (116) and the Human Gut Virome Database (6,360 viral populations; >10 kb) (34), or an additional 502 mouse gut virus genomes were curated (dereplicated into populations) from the IMG/VR database (117) and a published murine viral particle metagenome (118) (see Materials and Methods). Based on these comparisons, these murine gut viral species, which were previously unknown, expand the known murine gut viral sequence space ∼3-fold (Data Set S1, tab 13; see Materials and Methods).

At the genus level, gene-sharing network analyses (119) confidently placed 401 of the 1,028 SCI viral populations in the network as part of 163 viral clusters (VCs; equivalent to genus-level characterization). Of these, 89 VCs (containing 219 viral populations) were exclusive to our data set, whereas 74 VCs (containing 182 viral populations) included reference sequences that derive from human gut viruses and/or the NCBI RefSeq database (Data Set S1, tab 14, and Fig. S5A and B). Among these, only 58 murine gut viral populations could be assigned taxonomy (Fig. S5B), within the order *Caudovirales*, and the relative abundance of these phages was higher in the T4 SCI group (Fig. S5C). Previous studies in murine colitis models and human IBD patients also revealed a disease-dependent increase in *Caudovirales* (45, 118). These findings show that many of our murine gut viruses are unique at both the species and genus level and that murine gut viruses clustered more closely with human gut viral genomes than non-gut-derived viruses (Fig. S6A). Together, this is promising for preclinical SCI murine applicability toward human disease.

10.1128/mSystems.01356-20.6 FIG S5 *Caudovirales* phage abundances increased after spinal cord injury. (A) vConTACT 2.0 was used to construct a gene-sharing network (left) among three data sets: our SCI data set (*n* = 992), RefSeq prokaryotic viral genomes, v88, and the Human Gut Viral Database (GVD) (34, 116). Nodes (circles) represent genomes and contigs, and edges (lines) indicate shared protein content. (B) The pie chart (right) shows the vConTACT 2.0 summary of our 992 viral populations (>10 kb), which belong to “viral cluster, VC” (a group of sequences sharing more genes than others), “singleton” (single-member cluster having no shared gene content with other sequences), or “outlier” (weakly connected with a cluster of sequences). VCs are further divided into those without gene sharing with public databases “previously unknown VC,” those with gene sharing with public databases but without taxonomic affiliation, “unclassified, shared VC,” and those with taxonomic affiliation, “taxonomically classified VC.” (C) Box plot showing the median and quartiles of mapped read abundances represented by RPKM for the taxonomically classified viral populations (>10 kb) which were assigned to the *Caudovirales* phage order. Five individual mouse samples are used in each group. * *P* < 0.05 (by Wilcoxon signed-rank test, FDR < 0.1 calculated by the Benjamini and Hochberg method). The gene-sharing network was visualized by Cytoscape_v3.7.1. Download FIG S5, PDF file, 2.9 MB. Copyright © 2021 Du et al. 2021 Du et al. https://creativecommons.org/licenses/by/4.0/ This content is distributed under the terms of the Creative Commons Attribution 4.0 International license.

10.1128/mSystems.01356-20.7 FIG S6 Prediction of temperate phages. (A) An example of a confident prophage (phage NODE_5094) prediction, annotated and drawn by PHASTER. att, attachment sites; int, integrase; PLP, phage-like protein; Hyp, hypothetical protein; Ter, terminase; Por, portal protein; Coa, coat protein. (B) Percent of viral contigs (>5 kb) predicted as temperate phages using PHASTER and other different lysogeny marker genes, as listed on the *x* axis. Download FIG S6, PDF file, 1.0 MB. Copyright © 2021 Du et al. 2021 Du et al. https://creativecommons.org/licenses/by/4.0/ This content is distributed under the terms of the Creative Commons Attribution 4.0 International license.

**FIG 5:**
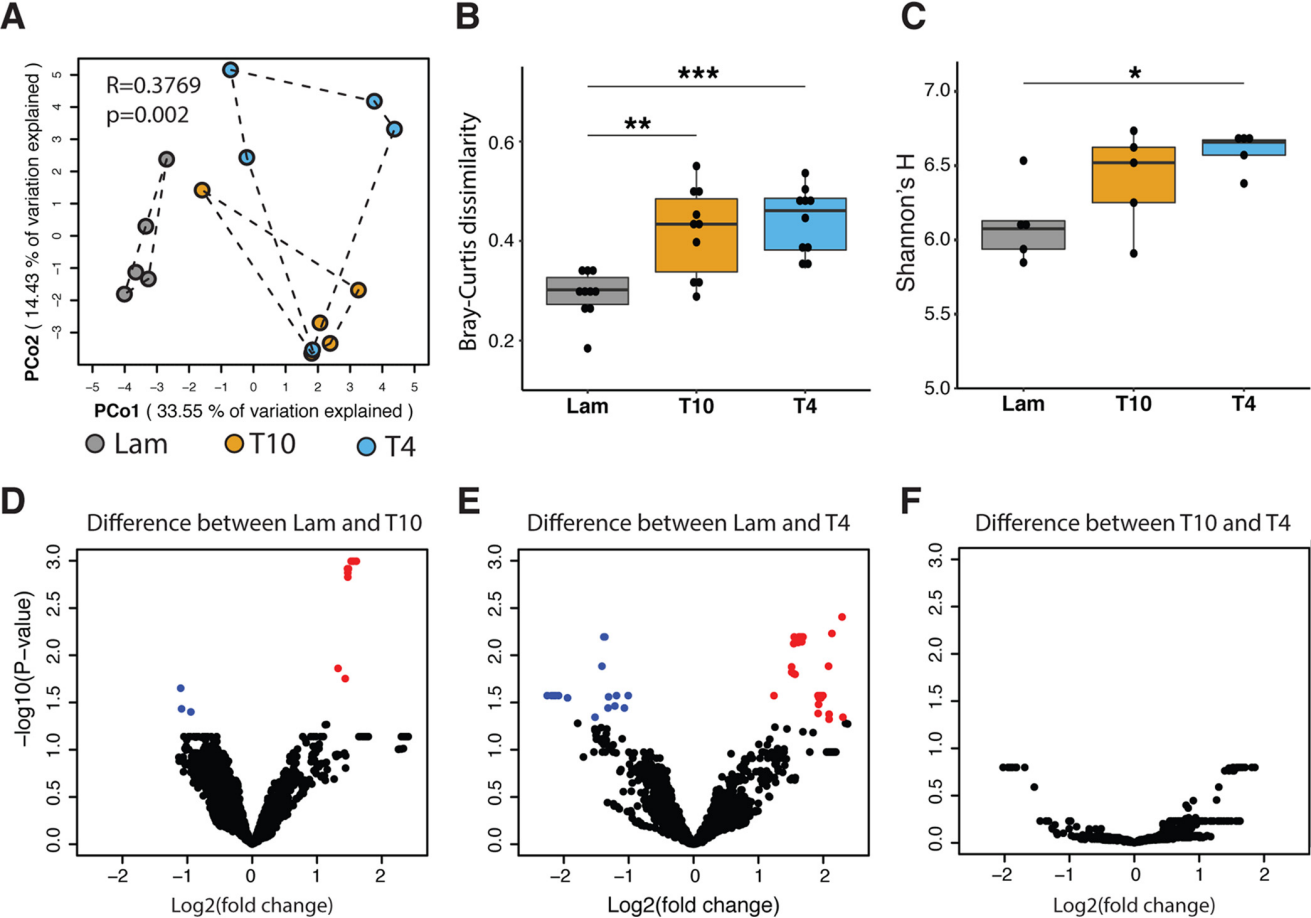
Viral communities are altered after SCI. (A) Principal-coordinate analysis (PCoA) of Bray-Curtis distances showing that viral communities are different between the Lam, T10, and T4 groups (analysis of similarity [ANOSIM], *P* = 0.003). Each data point represents an individual mouse sample (*n* = 15). (B) Box plot analysis showing the within-group Bray-Curtis dissimilarities in Lam, T4, and T10 viral communities. A higher score suggests higher dissimilarity of different samples in the same group. ***, *P* < 0.001; **, *P* < 0.01 (by Wilcoxon rank sum test with a false discovery rate [FDR] of <0.05 calculated by “fdr” in R). (C) Box plot analysis showing Shannon’s *H* of the viral communities between the Lam, T4, and T10 groups. Shannon’s *H* is an index of diversity, and a higher Shannon’s *H* suggests higher diversity of viral populations in the communities. All box plots shown display the median and quartiles, with each dot in the box plot representing an individual mouse sample, and each group (Lam, T4, T10) contains five samples. *, *P* < 0.05 (by Wilcoxon rank sum test with an FDR of <0.05). (D to F) Volcano plots of *t* tests corrected by the Benjamini and Hochberg method for changes to viral population abundances after SCI. An FDR cutoff of 0.05 was used. Data points highlighted in red indicate viral populations that were significantly enriched in T10 or T4 mice, while data points highlighted in blue indicate viral populations that were significantly enriched in the Lam mice.

With this reference data set in-hand, we next assessed the relative abundance of viral populations via nonredundant read mapping. As with the bacterial component of the gut microbiome, principal coordinate analysis (PCoA) of the gut virome (viral contigs ≥ 5 kb) revealed marked separation between the Lam and SCI groups (PERMANOVA, *R* = 0.3769, *P ≤ *0.002) (Fig. 5A). Moreover, gut viral within-group community dissimilarity was greater in SCI groups (Wilcoxon rank sum test, *P < *0.05, FDR < 0.05) (Fig. 5B), and viral species diversity was significantly increased in a spinal level-dependent manner with greater diversity in the T4 SCI group (Wilcoxon rank sum test, *P < *0.05, FDR < 0.05) (Fig. 5C). Viral diversity also increases in obesity and inflammatory bowel disease (45, 120, 121), indicating that SCI-induced changes in the gut virome may be associated with an inflammatory disease state. Further analyses of changes in viral population abundances among the groups revealed a relationship between SCI level and the number of viral populations (>5 kb) affected by SCI, as significant changes were noted in 21 and 57 viral populations in T10 and T4 SCI samples, respectively (*t* test adjusted, FDR *P* < 0.05) (Fig. 5D; Data Set S1, tab 15).

We next sought to assign infection types (lytic versus temperate) to the fuller data set of 2,675 viruses (>5 kb). To do this, we analyzed the contigs for prophage-bacterium junctions (attachment sites) (122) and signatures (integrase/site-specific recombinase, excisionase, repressor/antirepressor, and *parA*/*parB*) (123–126) of temperate phages (see Materials and Methods). This suggested that at least 516 contigs (∼19.3%) contained such signatures and so might be considered candidate temperate phages; 49 of these were more confidently temperate phages, as they contained identifiable putative attachment sites and integrase genes (Fig. S6B and Data Set S1, tab 16). These findings are similar to previous studies that established that temperate phages constitute about 20 to 50% of phages in the human gut (42, 127, 128). If we restricted these analyses to the 70 viral populations (>5 kb) that were differentially affected by SCI (Fig. 5D and E), 17 were identified as candidate temperate phages, with 3 confident temperate phages containing both identifiable attachment sites and integrases (Data Set S1, tab 17). Prior to these analyses, the effects of SCI on the virome had not been considered. These novel data indicate that SCI alters the gut virome, with the magnitude of effect being more severe after high-level SCI, impacting both temperate and lytic phages.

**FIG 6:**
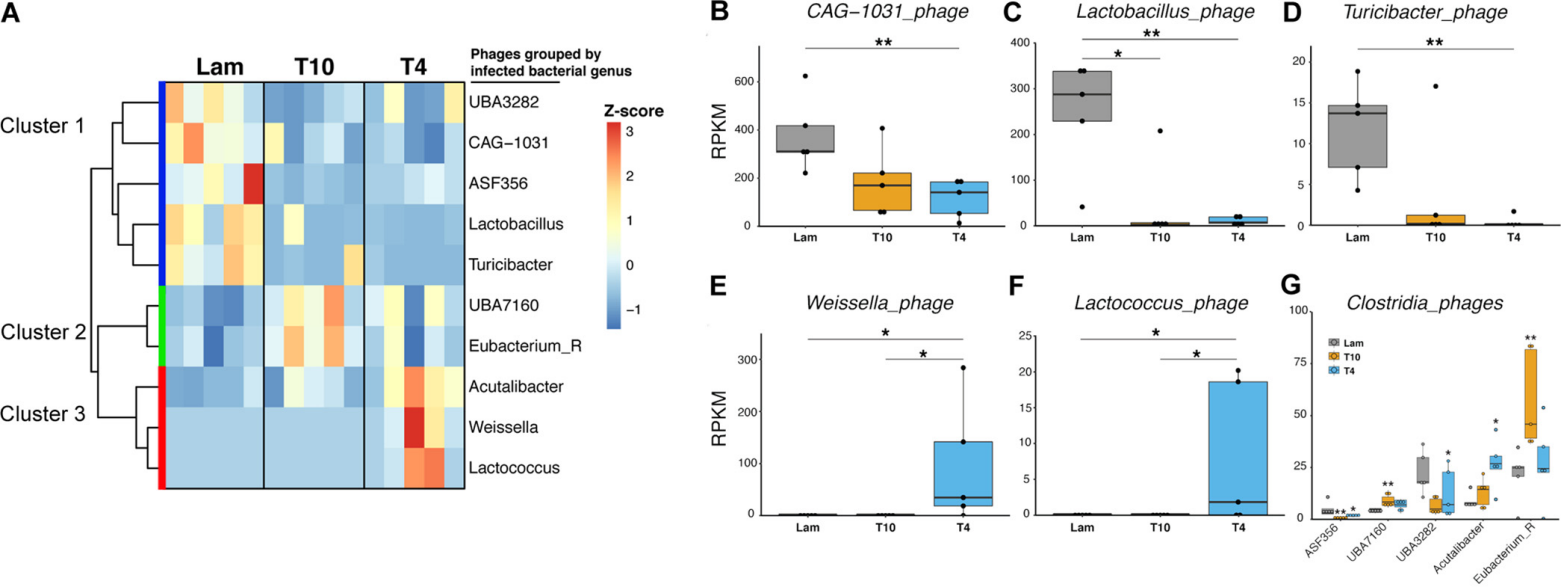
Viral host prediction reveals that phage abundances vary with their hosts. (A) Hierarchical clustering of these differently abundant phages (*P* < 0.05 by Wilcoxon rank sum test, FDR < 0.05) shows three distinct clusters. (B to D) Box plot analyses of select groups of phages are shown. Phages that were predicted to infect *CAG-1031*, *Lactobacillus*, and *Turicibacter* genera decreased after spinal cord injury. (E to F) Phages that were predicted to infect *Weissella* and *Lactococcus* increased after spinal cord injury. All box plots shown display the median and quartiles, with each dot in the box plot representing an individual mouse sample. Five individual mouse samples were used in each group. (G) Selected genus-specific phages constituting lower taxonomic ranks of the class *Clostridia*. **, *P* < 0.01; *, *P* < 0.05 (by Wilcoxon rank sum test). An FDR of <0.05 calculated by “fdr” in R was used here.

To develop hypotheses about how SCI-induced virome changes might impact the microbiome, we next sought to link viral contigs to microbial hosts (MAGs) via commonly used *in silico* approaches (similarity to host k-mer signatures [129], prophages [113], tRNAs [118, 130], and CRISPR spacers [130, 131]) (see Materials and Methods). These analyses predicted hosts for 35.5% of the viral contigs (Fig. S7 and Data Set S1, tab 18), which is three times higher than a previous murine gut virome analysis where only reference microbial genome databases were available (118) but on par with findings in soils where cosampled MAGs were also available (113). Using these prediction models, specific phages were significantly altered by SCI (Fig. 6; Fig. S6, Wilcoxon rank sum test, *P < *0.05, FDR < 0.05, and Data Set S1, tab 19). Hierarchical clustering of the differentially abundant phages again revealed 3 distinct clusters (Fig. 6A). Cluster 1 identified phages that were less abundant in both SCI groups compared to Lam controls. Phages in clusters 2 and 3 were more abundant in all SCI samples and T4 SCI samples, respectively, than in Lam controls (Fig. 6A). As with their bacterial hosts (Fig. 3), the relative abundances of phages that infect *Lactobacillus*, *CAG-1031*, and *Turicibacter* were reduced after SCI (Wilcoxon rank sum test, *P < *0.05, FDR < 0.05) (Fig. 6B to D; Fig. S6), whereas those predicted to infect *Weissella*, *Lactococcus* (Wilcoxon rank sum test, *P < *0.05, FDR < 0.05) (Fig. 6E and F) and class *Clostridia* increased after SCI (Wilcoxon rank sum test, *P < *0.05, FDR < 0.05) (Fig. 6G). These data illustrate the value of using cosampled microbial reference genomes (i.e., from the same bulk metagenomes) to make predictions about virus-host interactions. The fact that phage abundance patterns after SCI correspond with changes in microbial host abundance (Fig. 3) indicates that phages may also serve as biomarkers of systemic disease or neurological recovery after SCI and possibly in other disease states.

10.1128/mSystems.01356-20.8 FIG S7 Virus-host predictions. (A) A pie chart showing that 35.56% of the viral populations (≥5 kb) in this study were bioinformatically linked to a host. (B) Number of viral populations whose hosts can be predicted using different bioinformatic approaches, as listed on the *x* axis. Download FIG S7, PDF file, 0.8 MB. Copyright © 2021 Du et al. 2021 Du et al. https://creativecommons.org/licenses/by/4.0/ This content is distributed under the terms of the Creative Commons Attribution 4.0 International license.

Though the study of viruses in complex communities and viral ecogenomic approaches are in their infancy, particularly for inferring phage lifestyle (e.g., lytic or temperate), understanding phage-host dynamics is likely essential for us to reveal any causal roles played by the gut virome in disease (132). Lytic phages metabolically reprogram their bacterial hosts during infection in ways that alter that host’s output into the ecosystem (133) and ultimately kill their hosts to modulate host abundance and diversity through predator-prey dynamics (reviewed in reference 35). Temperate phages integrate themselves into host genomes and can regulate host gene function (reviewed in reference 134) and/or provide their hosts with new functions, like antibiotic resistance, toxin production, and other functions that may promote the virulence of commensals or confer fitness and competitive advantages to the hosts (123, 135). However, stressors, like antibiotics, hydrogen peroxide, and changes in nutrients and pH, which activate the bacterial host's SOS response, can induce temperate prophages, causing them to become lytic and ultimately kill host cells (123, 136, 137). Dietary fructose and short-chain fatty acids (SCFAs) were shown to induce Lactobacillus reuteri temperate phages (138), and bile salts were shown to induce some *Salmonella* temperate phages (139). Gut inflammation can also increase temperate phage induction in mice (140). Thus, a change in gut phage ecology after SCI, due to actions by both temperate and lytic phages, could dramatically influence how individuals respond to dietary changes and repeated regimens of antibiotics or drug therapies. In turn, these changes could have significant implications for recovery of function and the development of various comorbidities after SCI.

### Conclusions.

The gut microbiome has emerged as an essential component of human development, metabolism, and health, and growing evidence from gene marker data for microbes (1–4, 15) and metagenomic data for microbes and viruses (this study) suggest that this is also true for SCI. It is now clear that key probiotic bacterial populations and genes controlling their physiological functions are lost after SCI, suggesting that repopulating the gut with distinct bacterial taxa, such as Lactobacillus johnsonii and *CAG-1031* spp., may help to improve a range of functional outcomes after SCI. Also, SCI-induced changes in the gut microbiota may be influenced by corresponding changes in the gut virome, a previously uncharacterized mechanism associated with gut dysbiosis after SCI. These novel phage-host interactions could influence clinical outcomes and also serve as therapeutic targets. For example, the century-old idea of “phage therapy,” whereby bacterial viruses (or phages) are used to selectively target host pathogens, could represent a novel approach to treat bacterial infections, thereby reducing the need for antibiotics (141), which are variably effective and also harm commensal bacteria. By leveraging the expanding array of microbiome capabilities and resources (142–144) and the partnered viral ecogenomic toolkits for capture (145, 146) and characterization (52–54), it is now possible to establish novel metagenome-enabled hypotheses regarding the effects of SCI on gut microbial ecology and how those changes can functionally influence mammalian physiology.

## MATERIALS AND METHODS

### Animals and spinal cord injury.

All surgical and postoperative care procedures were performed in accordance with the Ohio State University Institutional Animal Care and Use Committee. Fifteen female C57BL/6 mice from Jackson Laboratories (Bar Harbor, Maine) were used in this study. To minimize external/environmental effects on the microbiota, we were meticulous in our efforts to establish controlled conditions, as follows. All animals were ordered together and arrived in the same cohort from Jackson Laboratories. To prevent gut microbial cross-contamination due to cohabitation, all mice were singly housed upon arrival at our animal facility and for the duration of the study in a vivarium room that contained no other animals. All mice remained in this same room for the duration of the study. No mice received antibiotics or dietary supplements at any point throughout the study. Quantity of food intake was not measured, but all mice received the same standard rodent chow for the duration of the study. Our data show that the effects of SCI are consistent (only one outlier was noted using multivariate statistics; see Fig. 1B as an example) and robust. Moreover, there are clear spinal level-dependent differences.

Mice were anesthetized with an intraperitoneal cocktail of ketamine (80 mg/kg)-xylazine (10 mg/kg), after which a partial laminectomy was performed at the 4th thoracic spine (T4) or the 10th thoracic spine (T10). To create consistent severe SCI in each mouse, the spinal cord located between the T3 and T4 or T10 and T11 vertebrae was crushed by inserting modified no. 5 Dumont forceps (Fine Science Tools; with a tip of 0.4 to 0.2 mm) 2 mm ventrally into the vertebral column on both sides of the spinal cord and then laterally compressing the spinal cord by bringing the forceps tips together completely, so they are touching for 3 s. This lesion leaves the dura intact but creates a severe lesion with minimal sparing of ascending or descending axons in the white matter (147). Postoperatively, animals were hydrated with 2 ml Ringer’s solution (subcutaneous) for 5 days. Bladders were voided manually at least twice daily for the duration of the study. No prophylactic antibiotics were used during or after surgery. Fecal samples were collected 21 days postinjury (dpi). Mice were removed from their home cage and placed into a ventilated, aseptic polystyrene compartment, and fresh fecal samples were collected from each mouse into sterile tubes and immediately frozen in liquid nitrogen. Mice were returned to their home cage after sample collection. In total, five mice received a complete crush at T4, five mice received a complete crush at T10, and five mice did not receive any crush after laminectomy (Lam group). In our previous work (19), we collected serial samples from the same Lam mice and showed that laminectomy alone (control for both anesthesia and surgery) did not cause differences in gut microbiota when analyzed using 16S rRNA gene sequencing.

### Ethics approval and consent to participate.

The Institutional Animal Care and Use Committee of the Office of Responsible Research Practices at The Ohio State University approved all animal protocols. All experiments were performed in accordance with the guidelines and regulations of The Ohio State University and are outlined in the *Guide for the Care and Use of Laboratory Animals* from the National Institutes of Health.

### Metagenomic sequencing, read quality control, and contigs assembly.

Bulk DNA was recovered from the 15 fecal samples separately using a ZymoBIOMICS extraction kit. Metagenomic library preparation and shotgun sequencing were conducted at CosmosID using an IonTorrent Ion S5 next-generation sequencing system. On average, 23.5 million single-end reads were generated per sample (range, 15.2 million to 36.2 million reads), with an average read length of 180 bp. Reads were quality trimmed using bbduk (https://jgi.doe.gov/data-and-tools/bbtools/) from both ends to remove bases with low-quality scores (qtrim = rl, trimq = 10) and positions with high compositional bias (ftl = 10, ftr = range from 204 to 229 depending on the sample). Reads shorter than 30 bp (minlength = 30), with Ns (maxns = 0) or with an average quality below 10 (maq = 10) were discarded. Mouse reads were removed from all the samples using bbmap (https://jgi.doe.gov/data-and-tools/bbtools/) by mapping against the genome of our model mouse strain C57BL/6NJ (downloaded from the NCBI assembly database; GCA_001632555.1) and removing reads with a minimum identity of 95% (minid = 0.95). After quality control, all the clean single-end reads were cross-assembled using SPAdes (v3.11.1) (148) in the “read-error correction and assembling” mode and using the (-iontorrent) flag. The full k-mer size list (-k 21, 33, 55, 77, 99, 127) was used in the assembly. All bioinformatic analyses were performed within the Ohio Supercomputer Center (149). For a visual overview of the bioinformatic analyses, see Fig. S1A in the supplemental material.

### Read-based estimation of microbial diversity and community structure.

Reads from each sample were piped through SingleM (singlem pipe; https://github.com/wwood/singlem) to estimate the abundance of discrete taxa down to the strain level (*Sensu* [150]). Relative abundances of taxa, which were used for all phylum- and genus-level comparisons, were calculated from the mean coverage of 14 single-copy marker genes to avoid copy number variations associated with 16S-based estimates of abundance and to increase taxonomic resolution. Abundances of taxa inferred from the coverage of ribosomal protein L2 (rplB), which is widely used for microbial community analysis (151), were used for principal-coordinate analysis of Bray-Curtis dissimilarity. Within- and between-group Bray-Curtis dissimilarity analyses were performed using vegan in R. Principal-coordinate analysis (function capscale with no constraints applied) was carried out on the Bray-Curtis dissimilarity matrix (function vegdist; method “bray”) after a log_2_ transformation on the relative abundance matrix. A pseudo count of 1 was added to all the cells before this transformation to avoid negative numbers. The three groups that emerged in the ordination plot were tested using a PERMANOVA test (function “anosim”) and were defined on the plot using function “ordihull.” For a visual overview of the bioinformatic analyses, see Fig. S1B.

### Construction of MAGs, estimation of abundance, and taxonomic classification.

Microbial metagenome-assembled genomes (MAGs) were recovered using the coassembly-optimized tool MaxBin 2.0 (v2.2.4) (152), which depends on the tetranucleotide frequencies of the contigs, a phylogenetic marker gene set, and differential coverage binning. First, all reads from each sample were mapped to the coassembled contigs using Bowtie2 (v2.3.4.1) (148). The number of mapped bases (for average coverage calculation) and reads (for calculation of normalized reads per kilobase per million mapped reads [RPKM]) were counted using BEDTools (v2.23.0) (153) in “mapbamsamples.pl” of SqueezeMeta (May 2018 distribution) (154). The MAGs were then binned by MaxBin 2.0 using each sample’s contigs’ coverage (-abund_list) and the full marker gene set. CheckM (v1.0.12) (155) was then used to assess the quality (completeness and contamination) of the genome bins using the “lineage_wf” pipeline, and genome bins were filtered at a completeness of ≥60% and a contamination of ≤10%. dRep (v2.2.3) (156) was used then to dereplicate the MAGs at 97% average nucleotide identity (dRep_97 -comp 60 -con 10 -sa 0.97). After the dereplication, 112 MAGs were recovered (>60% complete, <10% contamination), including 70 high-quality MAGs (>90% complete, <5% contamination) and 35 medium-quality MAGs (>70% complete, <10% contamination) that were recovered from our 15 samples. After the recovery of the MAGs, GTDB-Tk (v0.1.3) (63) was used to assign taxonomic classifications for the 112 MAGs in the “classify_wf” mode. In total, 9 MAGs can be confidently resolved into bacterial species. In addition, among the rest of the MAGs, 73 MAGs can be assigned to bacterial genera. The coverm (https://github.com/wwood/CoverM) was conducted for mapping the reads in different samples to the reference genome (MAGs) in “genome” mode and “single” parameter. Data Set S1 describes the characteristics of the dereplicated MAGs and the predicted taxon of each MAG. For a visual overview of the bioinformatic analyses, see Fig. S1B.

### Identification of viral contigs and establishing viral populations from the bulk metagenomes.

With a high sequencing depth (average of 23.5 million reads/sample compared to ∼5 to 10 million in more typical gut microbiomes [157]) in the bulk metagenomes, we were able to identify putative viral contigs, including a mix of actively infecting lytic viruses and prophages, from the bulk metagenomes following assembly. An ensemble approach was used where, first, all contigs were analyzed using VirSorter (v1.0.5) (158), DeepVirFinder (v1.0) (159), MARVEL (v0.1) (160), and CAT (https://github.com/dutilh/CAT). This approach combines homology-based identification (CAT and VirSorter), sequence composition in deep learning (DeepVirFinder), and genomic features in probabilistic models (VirSorter and MARVEL). VirSorter was used in the “bulk metagenome” mode and with selection of the virome database, while DeepVirFinder was allowed to predict contigs down to 300 bp in length. Next, linear contigs of ≥5 kb and circular contigs of  ≥1.5 kb that were sorted as VirSorter categories 1 to 6, by a DeepVirFinder score of  ≥0.7 (*P* value < 0.05), and/or by a MARVEL random forest probability of  ≥70% were kept for further investigation. Of these contigs, those that were sorted as VirSorter categories 1, 2, 4, and 5, by a DeepVirFinder score of  ≥0.9, or by a MARVEL probability of ≥90% were considered viral. For the rest of the kept contigs, they were considered viral only if they were identified by at least two tools, VirSorter (categories 3 or 6), DeepVirFinder (score of  ≥0.7 and <0.9), MARVEL (probability of ≥70% and <90%), and/or CAT (annotated as viral or <40% of the genes were classified nonviral). In total, 29,143 viral contigs were identified, with 2,675 viral contigs of ≥5 kb and 1,030 viral contigs of ≥10 kb. Then, the identified putative viral contigs were clustered into viral populations using Clustergenomes (v1.1.0; https://bitbucket.org/MAVERICLab/stampede-clustergenomes/src/master/) at ≥95% nucleotide identity across ≥80% of the shorter genome length (55). This resulted in 2,658 viral populations of ≥5 kb and 1,028 viral populations of ≥10 kb (see Data Set S1, tab 20, for VirSorter, Deep VirFinder, MARVEL, and CAT results). Notably, the number of viral populations of ≥5 kb recovered from the 15 samples in this study significantly exceeds the global average of 1,581 viral populations per study at the same contig length cutoff (34). Clustergenomes analysis was also conducted using a combination of our 1,028 viral populations with viral populations (>10 kb) in public databases, including NCBI RefSeq v88 (116), the Human Gut Virome Database (34), and the murine gut virome from the IMG/VR database (117), and one murine viral particle metagenome (118) to determine the novelty of our viral species. For a visual overview of the bioinformatic analyses, see Fig. S1C.

### Viral taxonomy and virus-host predictions.

Viral genus-level taxonomy was assigned using vConTACT 2.0 (v2-0.9.9) (119) by clustering (–rel-mode BLASTP –pcs-mode MCL –vcs-mode ClusterONE) our 1,028 viral populations (>10 kb) with both the RefSeq viral genome database (v88) (4,061 complete genomes; >10 kb) (116) and the Human Gut Virome Database (6,360 viral populations; >10 kb) (34). The viral populations that can be clustered with a viral genome from RefSeq were likely to be assigned to a known viral taxonomic genus, family, or order classified by the International Committee on Taxonomy of Viruses (ICTV) taxonomy. Data Set S1, tab 14, summarizes the viral clusters using vConTACT 2.0. Four different computational methods were used to predict putative hosts for the viral populations, prophage-BLAST, CRISPR spacer matches, tRNA exact matches, and k-mer-based sequence similarity. For prophage-BLAST, the viral genome nucleotide sequences were compared to the MAGs using BLASTn based on a method described previously (161). To improve prediction accuracy, only hits with 100% identity over 100% of the length of viral contigs were used for further prediction. Only prophage-BLAST predictions that can be identified using PHASTER (162) were considered. For CRISPR linkages, MinCED (v0.2.0; https://github.com/ctSkennerton/minced) was used to search for prokaryotic CRISPR spacers (with a minimum of 2 repeats that a CRISPR must contain) from our set of MAGs. Then, the prokaryotic CRISPR spacers were matched to viral contigs using BLASTn. Only hits with at least 95% identity over the whole spacer length were considered. For tRNA linkages, tRNAscan-SE (v1.23) (163) was conducted to identify tRNAs from viral contigs (using the general model -G) and MAGs (using the bacterial model -B). Then, tRNA secondary structure sequences from viral contigs were compared to those from MAGs using BLASTn. Only hits with 100% identity over 100% of the length, and at least one hit in each MAG that was consistent with prophage-BLAST prediction, were considered. For k-mer frequency linkages, host-virus connections were predicted using WIsH (v1.0) (129). To assign *P* values to individual virus-host predictions, we built a null model using all the sequences in the RefSeq viral genomes database. Only WIsH prediction with a *P* of 0 and the consistent predictions from both WIsH with a *P* of <0.05 and prophage-BLAST were considered. Figure S7 and Data Set S1, tab 18, show the results from viral host prediction using different methods.

### Annotating genes and making protein clusters.

We annotated genes on all of the assembled contigs by first predicting the open reading frames (ORFs) using Prodigal (v2.6.3) (164) in the metagenomic mode (-p) and ignoring any masked non-protein-coding sequences (-m) produced by “barrnap.pl” of SqueezeMeta. Next, the ORFs were annotated using a pipeline described previously (165). Briefly, annotations were conducted by running a combination of reciprocal best blast hit searches against the KEGG (Kyoto Encyclopedia of Genes and Genomes) (166) and UniRef90 (167) databases in tandem with HMM searches against Pfams (168). About half of ORFs can be annotated using KEGG, and KEGG annotations were then used for downstream analyses. MMseqs2 (version 4eb5e14267f64f2fb337995bd824ef279e04f266) (169) was used for clustering of all the protein sequence (cluster –min-seq-id 0.3 –cov-mode 1 -c 0.7 -e 0.00001), and the abundances of all the proteins within each cluster were summed to represent the total abundance of each unique protein clusters (PCs). In total, 335,087 protein clusters were identified for PCoA analyses.

### Identification of candidate temperate phages.

To infer phage lifestyle, prophage-bacterium junctions (attachment sites) (122) and lysogeny signatures were searched to identify candidate temperate phages. Candidate identifiable prophages were first identified with PHASTER (162), followed by manual inspection of the annotations for the presence of the attL and attR attachment sites (att). Then, lysogeny signatures (123–126), including integrase(s)/site-specific recombinase, excisionase, phage repressor/antirepressor, and ParA/B genes, were searched by annotating genes of the 2,675 viral genomes (>5 kb) against the KEGG (166) and UniRef90 (167) databases in tandem with HMM searches against Pfams (168). A prophage usually inserts into the host genome by integrases, which mediate site-specific recombination between the phage attachment site (attP) and the bacterial attachment site (attB) (170, 171). This results in an inserted prophage flanked by two hybrid sites (attL and attR), also needed for the reverse reaction (excision of the phage from the chromosome) along with excisionases (170, 171). Thus, a viral contig can be considered a typical prophage region if it has an integrase HMM match and attachments sites (attL and attR). Figure S6A is a clear example of a “confident” temperate phage, with putative “att” sites next to an integrase gene in the noncoding region. A viral contig containing only lysogeny markers is considered a “candidate” temperate phage. Figure S6B and Data Set S1, tabs 16 and 17, summarize the results from PHASTER and lysogeny markers.

### Statistical analyses.

All the statistical analyses were conducted using RStudio. The Wilcoxon rank sum test was conducted using the function “wilcox.test” in R, and “pairwise.wilcox.test” with adjusted method “fdr” was used to calculate the false discovery rate (FDR). “edgeR” and “limma” in R were used for differential analysis for viral populations (>5 kb) in different treatments. The “ggplot2” R package was used for box plots and bar plots. The R package “VennDiagram” was used for Venn diagram plotting, and “pheatmap” was used for heat maps.

### Data availability.

Scripts used in this manuscript are available on the Sullivan laboratory bitbucket under SCI (https://bitbucket.org/MAVERICLab/spinal-cord-injury-ion-torrent-project/src/master/). Raw reads and processed data are available through CyVerse under iVirus folder, including all assembled contigs, microbial MAGs, and viral populations.

10.1128/mSystems.01356-20.9 FIG S8 Differential abundance analysis of host-linked phages across healthy and SCI animals. Heat map showing Z-score-normalized abundances of phages grouped by their predicted bacterial hosts. Differentially abundant phages (*P* < 0.05 by Wilcoxon signed-rank test, FDR < 0.05 calculated by the Benjamini and Hochberg method) in either of the two SCI groups are indicated in red. Each row representing a unique bacterial genus was Z-score normalized. Download FIG S8, PDF file, 0.5 MB. Copyright © 2021 Du et al. 2021 Du et al. https://creativecommons.org/licenses/by/4.0/ This content is distributed under the terms of the Creative Commons Attribution 4.0 International license.
