# Supplementary figures and images for: Spinal Cord Injury Changes the Structure and Functional Potential of Gut Bacterial and Viral Communities
Source: mSystems. 2021 May 11;6(3):e01356-20. doi: 10.1128/mSystems.01356-20 (PMC8125080; doi:10.1128/mSystems.01356-20)

A

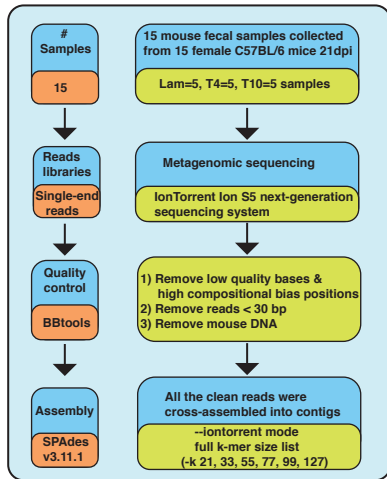

B

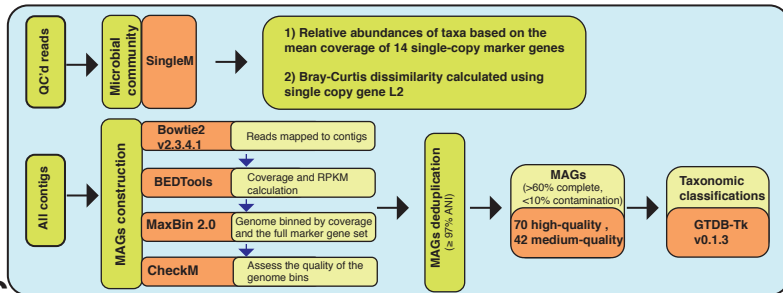

C

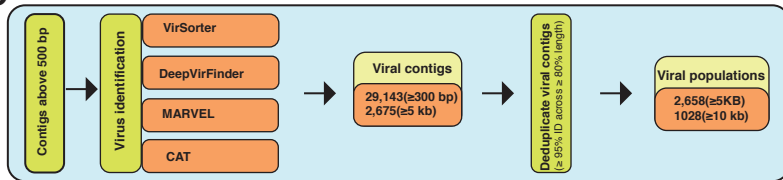

Supplement: FIG S1 [file mSystems.01356-20-sf001.pdf]

A

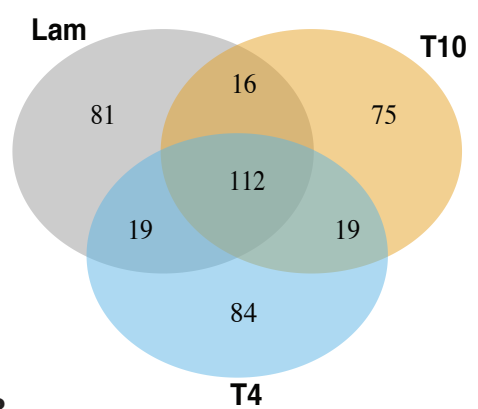

B

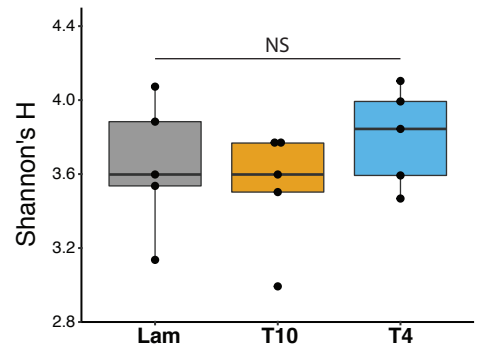

D

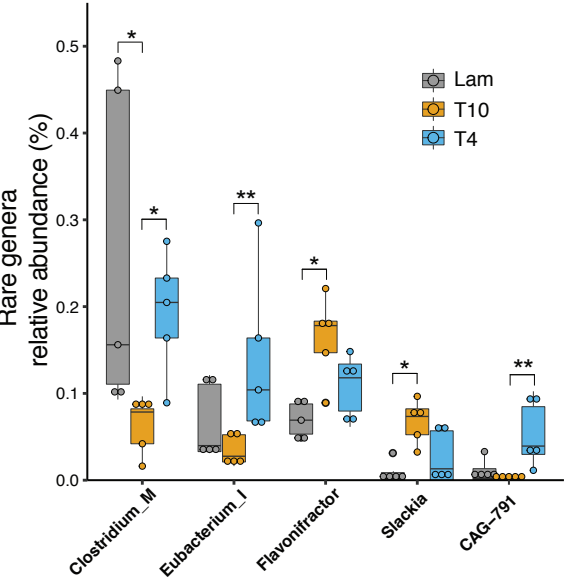

C

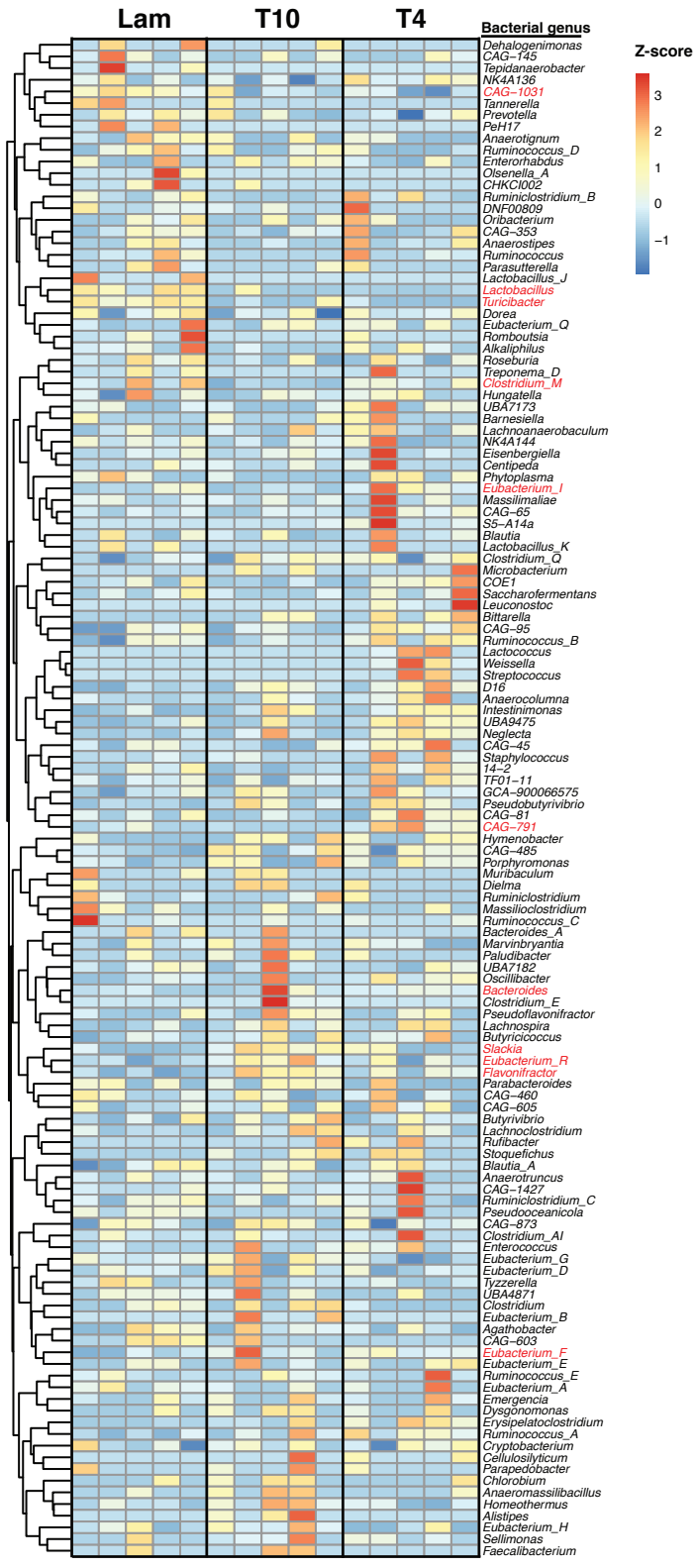

Supplement: FIG S2 [file mSystems.01356-20-sf002.pdf]

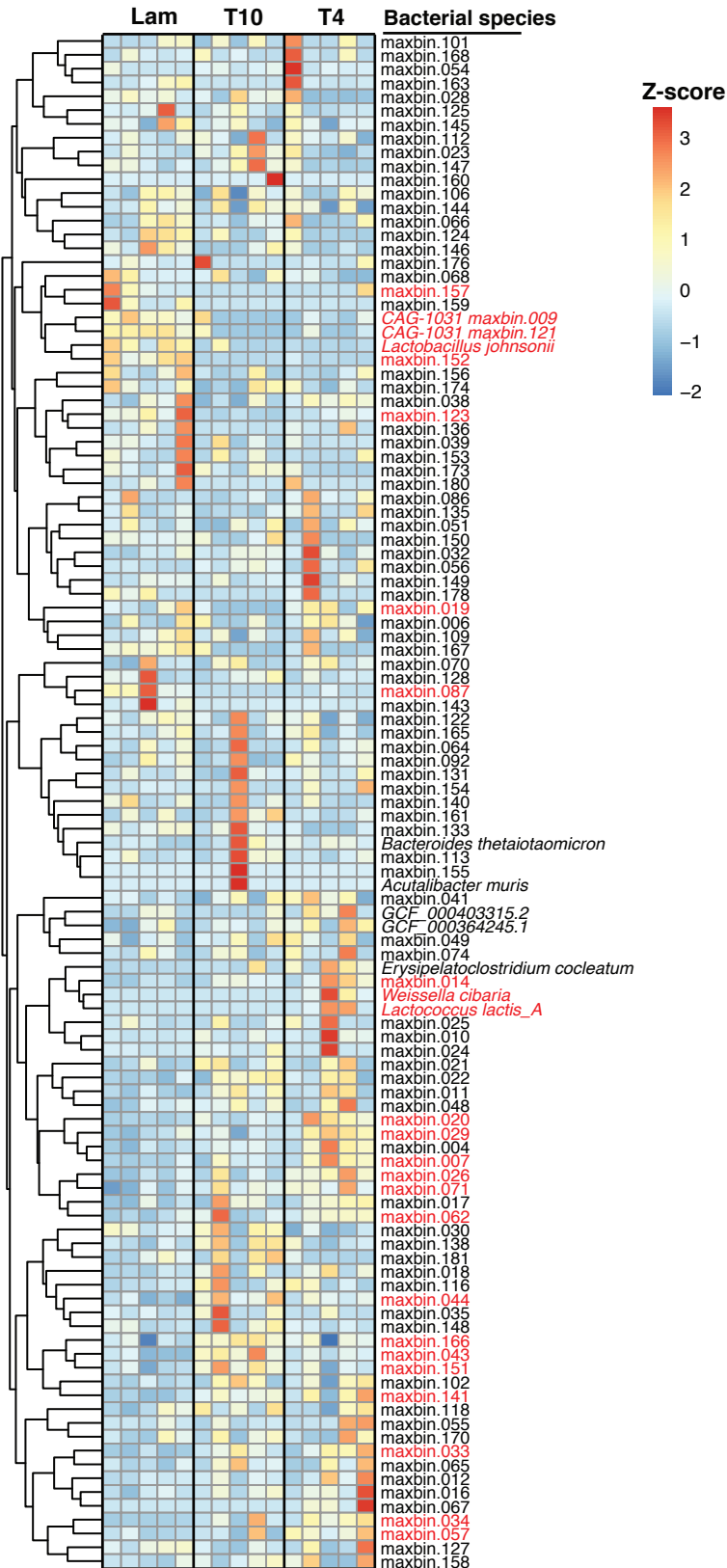

Supplement: FIG S3 [file mSystems.01356-20-sf003.pdf]

A

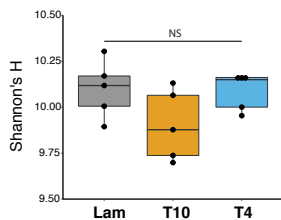

B

## Microbial Metabolisms

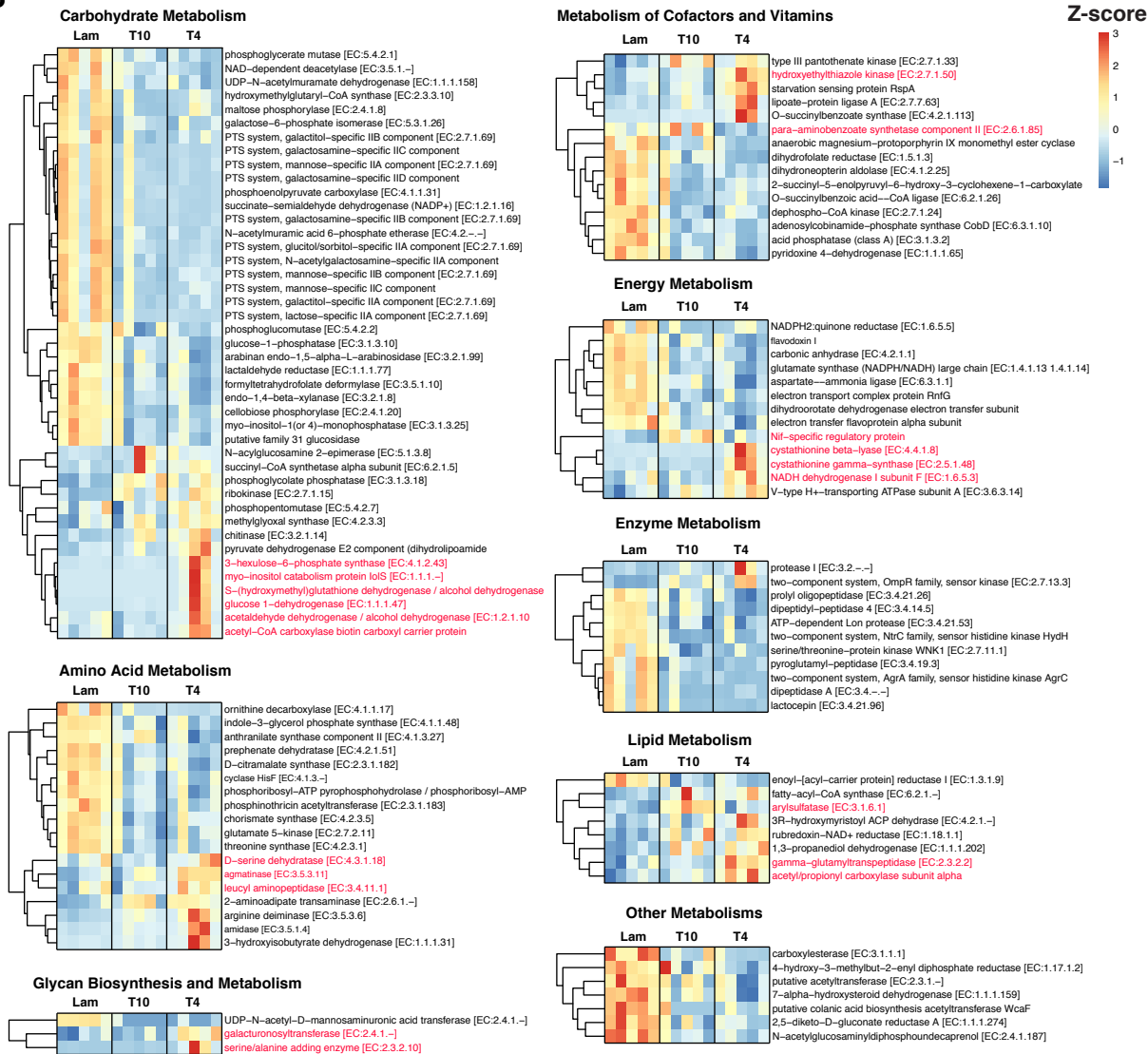

Supplement: FIG S4 [file mSystems.01356-20-sf004.pdf]

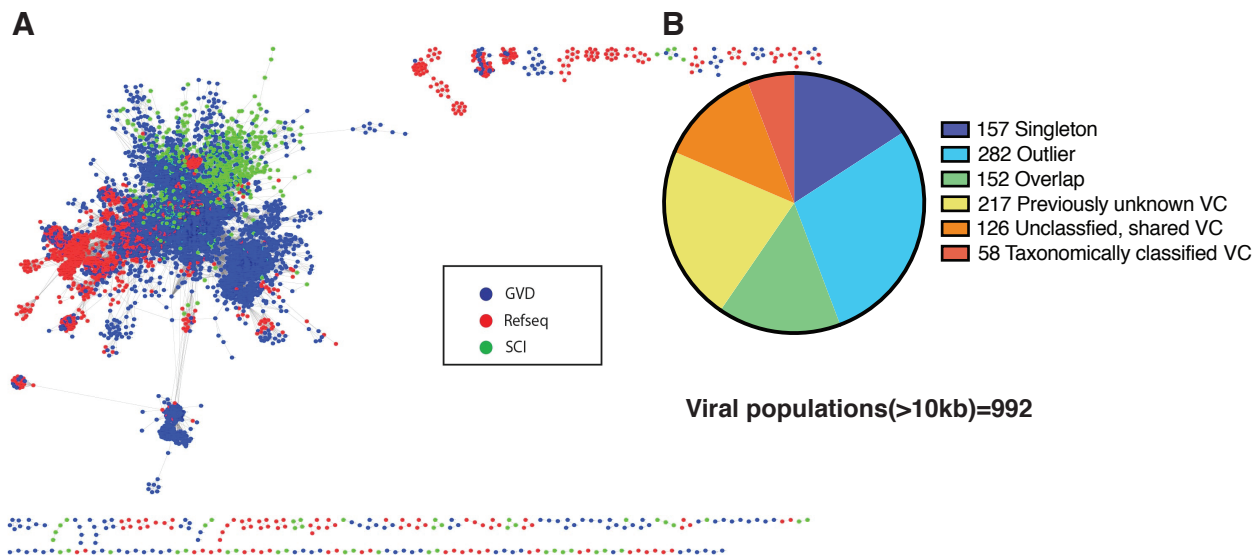

*Caudovirales*

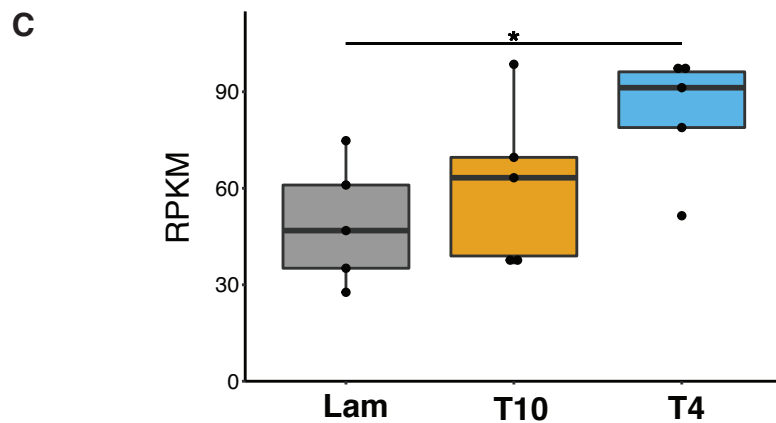

Supplement: FIG S5 [file mSystems.01356-20-sf005.pdf]

S6

A

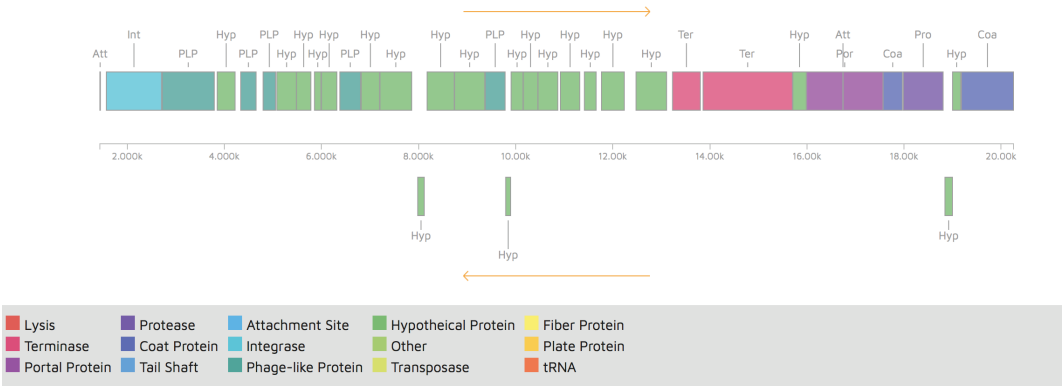

B

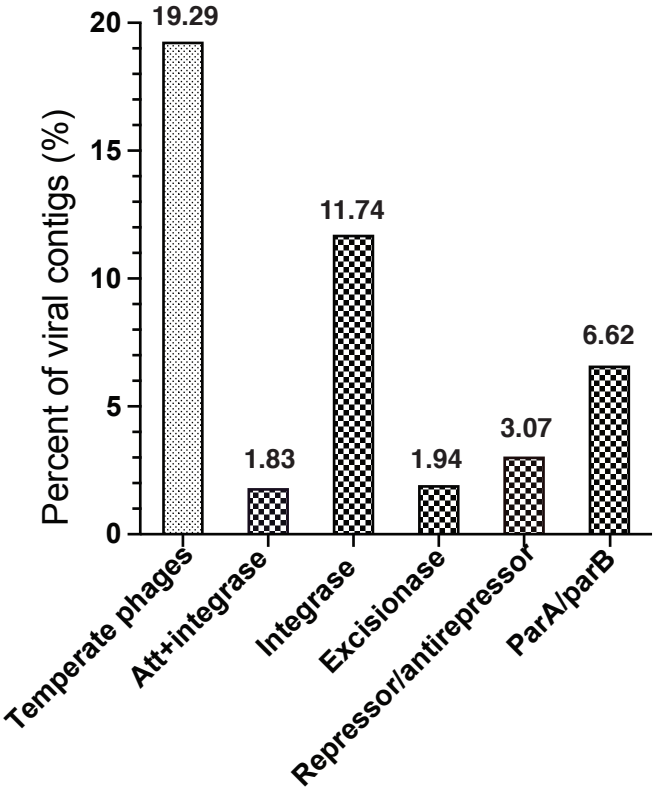

Supplement: FIG S6 [file mSystems.01356-20-sf006.pdf]

# S7

## A

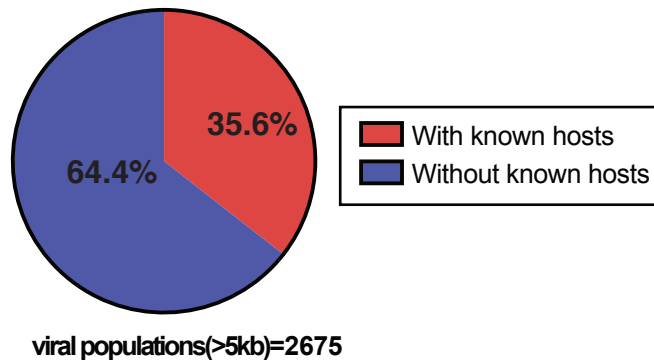

## B

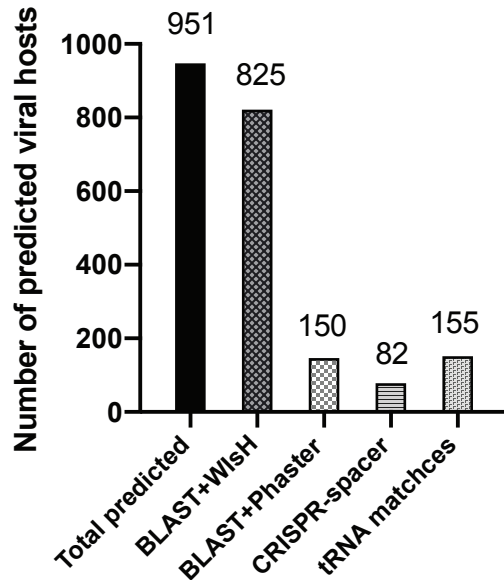

Supplement: FIG S7 [file mSystems.01356-20-sf007.pdf]

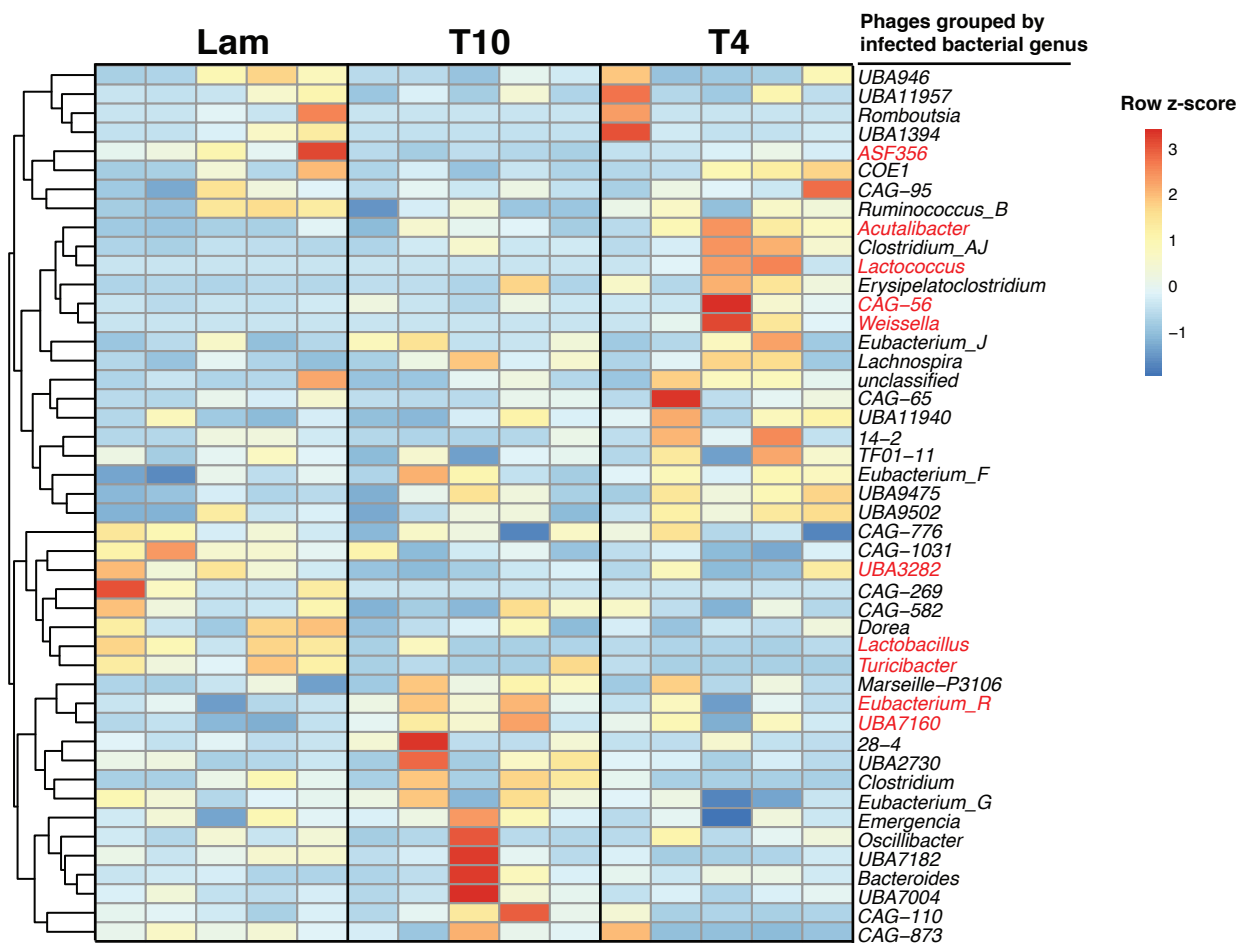

Supplement: FIG S8 [file mSystems.01356-20-sf008.pdf]
